# Optimal Detection of Fusion Pore Dynamics Using Polarized Total Internal Reflection Fluorescence Microscopy

**DOI:** 10.3389/fmolb.2021.740408

**Published:** 2021-11-10

**Authors:** Joerg Nikolaus, Kasey Hancock, Maria Tsemperouli, David Baddeley, Erdem Karatekin

**Affiliations:** ^1^ Cellular and Molecular Physiology, Yale University, New Haven, CT, United States; ^2^ Nanobiology Institute, Yale University, West Haven, CT, United States; ^3^ Integrated Physical and Engineering Biology Program, Yale University, New Haven, CT, United States; ^4^ Cell Biology, Yale University, New Haven, CT, United States; ^5^ Molecular Biophysics and Biochemistry, Yale University, New Haven, CT, United States; ^6^ Saints-Pères Paris Institute for the Neurosciences (SPPIN), Université de Paris, Centre National de la Recherche Scientifique (CNRS), Paris, France

**Keywords:** membrane fusion, SNARE-mediated membrane fusion, total internal reflection fluorescence microscopy, fusion pore, liposome-supported bilayer fusion assay

## Abstract

The fusion pore is the initial narrow connection that forms between fusing membranes. During vesicular release of hormones or neurotransmitters, the nanometer-sized fusion pore may open-close repeatedly (flicker) before resealing or dilating irreversibly, leading to kiss-and-run or full-fusion events, respectively. Pore dynamics govern vesicle cargo release and the mode of vesicle recycling, but the mechanisms are poorly understood. This is partly due to a lack of reconstituted assays that combine single-pore sensitivity and high time resolution. Total internal reflection fluorescence (TIRF) microscopy offers unique advantages for characterizing single membrane fusion events, but signals depend on effects that are difficult to disentangle, including the polarization of the excitation electric field, vesicle size, photobleaching, orientation of the excitation dipoles of the fluorophores with respect to the membrane, and the evanescent field depth. Commercial TIRF microscopes do not allow control of excitation polarization, further complicating analysis. To overcome these challenges, we built a polarization-controlled total internal reflection fluorescence (pTIRF) microscope and monitored fusion of proteoliposomes with planar lipid bilayers with single molecule sensitivity and ∼15 ms temporal resolution. Using pTIRF microscopy, we detected docking and fusion of fluorescently labeled small unilamellar vesicles, reconstituted with exocytotic/neuronal v-SNARE proteins (vSUVs), with a supported bilayer containing the cognate t-SNAREs (tSBL). By varying the excitation polarization angle, we were able to identify a dye-dependent optimal polarization at which the fluorescence increase upon fusion was maximal, facilitating event detection and analysis of lipid transfer kinetics. An improved algorithm allowed us to estimate the size of the fusing vSUV and the fusion pore openness (the fraction of time the pore is open) for every event. For most events, lipid transfer was much slower than expected for diffusion through an open pore, suggesting that fusion pore flickering limits lipid release. We find a weak correlation between fusion pore openness and vesicle area. The approach can be used to study mechanisms governing fusion pore dynamics in a wide range of membrane fusion processes.

## Introduction

Membrane fusion is a ubiquitous biological process required, e.g., for neurotransmitter and hormone secretion, infection of host cells by enveloped viruses, development, and fertilization (Chernomordik and Kozlov, 2008; Martens and McMahon, 2008). The initial connection between the apposed membranes is a small, ∼1 nm wide dynamic structure called the fusion pore. Fusion pore dynamics have been studied extensively for fusion of enveloped viruses (Spruce et al., 1989; Cohen and Melikyan, 2004; Harrison, 2008) and calcium-triggered secretion of hormones from neuroendocrine cells (Chang et al., 2017; Karatekin, 2018; Rorsman and Ashcroft, 2018; Sharma and Lindau, 2018). It was found that in both cases the fusion pores can flicker open-closed repeatedly at rates up to 4,000 Hz (Cohen and Melikyan, 2004; He et al., 2006; Klyachko and Jackson, 2002; de Toledo et al., 1993; Zhou et al., 1996; Staal et al., 2004; Melikyan et al., 1995; Melikyan et al., 1993a; Melikyan et al., 1993b), then either dilate further or reseal (Monck and Fernandez, 1994; Lindau and Alvarez de Toledo, 2003; Jackson and Chapman, 2008; Karatekin, 2018; Sharma and Lindau, 2018). For neurons, direct measurements of fusion pores are not as abundant but available measurements suggest there is large diversity in fusion pore dynamics, with a clear contribution to release kinetics or endocytosis in some cases (Verstreken et al., 2002; Gandhi and Stevens, 2003; Pawlu et al., 2004; Staal et al., 2004; He et al., 2006; Lisman et al., 2007; Alabi and Tsien, 2013; Chapochnikov et al., 2014). Fusion pores can also act as size-selective filters, as small cargo molecules can escape through a narrow pore while larger cargo are retained (Barg et al., 2002; Hastoy et al., 2017; Rorsman and Ashcroft, 2018). Invasion by enveloped viruses requires the fusion pore to dilate sufficiently to allow the release of viral genetic material into the host (Cohen and Melikyan, 2004). Despite clear evidence that pore flickering occurs, and that the temporal evolution of the fusion pore is a critical determinant of release kinetics and membrane recycling pathways, the mechanisms are poorly understood, partly due to a lack of assays with the required sensitivity and time resolution.

**FIGURE 1:**
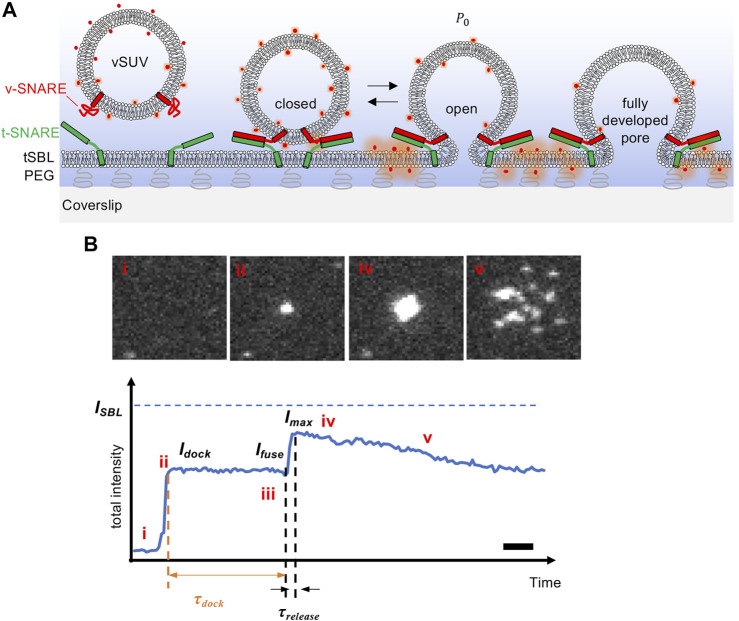
Intensity changes during liposome-supported bilayer fusion, detected using TIRF microscopy with s-pol excitation. **(A)** Schematic of the experiment. Small unilamellar vesicles reconstituted with neuronal/exocytic v-SNARE proteins (vSUVs) are labeled with a fluorescent lipid whose excitation dipole is nearly parallel to the membrane. The vSUV docks onto and fuses with a planar bilayer supported on a coverslip, and reconstituted with cognate t-SNAREs (tSBL). The membranes contain 5 mol % PEGylated lipids in order to avoid direct contact of the SBL with the coverslip. Only PEGylated lipids under the SBL are shown for clarity. Once a fusion pore is established, it can flicker open-closed (spending a fraction $P_{o}$ of the time in the open state), before eventually expanding or resealing. During fusion, the total fluorescence intensity around the fusion site increases as the fluorophores are transferred into the SBL, because the fluorophores are excited more efficiently in the SBL than the SUV. **(B)** An example of a vSUV-tSBL fusion event. Top: Snapshots extracted from an image stack recorded at 56 Hz. Every box is 11 μm -by- 11 μm (43 pixels by 43 pixels). Bottom: the total intensity (sum of pixel values) in a 22 μm -by- 22 μm box centered around the docking site, as a function of time, for the event shown above. vSUVs were labeled with 1 mol % LR-PE and excited at 561 nm in TIRFM. Because the evanescent field is very shallow, vSUVs that are ≳ 100 nm away from the SBL are not visible. Upon initial docking, a fluorescent spot appears and the integrated intensity rapidly increases to a new value (i–ii). Membrane fusion causes a rapid increase in intensity to $I_{max}$ (iii–iv), which is < than the values $I_{SBL}$ that would be reached were there no photobleaching. After sufficient time, individual lipid-linked fluorophores become visible as they disperse and photobleach (v). The box is chosen large enough that no fluorophores have yet left the box. Fitting the intensity trace to a model, the parameters shown on the figure are extracted. Scale bar = 200 ms.

Reconstitution has been key to understand basic mechanisms of the membrane fusion process (Rothman, 2014), with recent applications increasingly focusing on fusion pores (Karatekin, 2018). Where direct measurements are challenging, such as in neurons for monitoring fusion pore dynamics, or for intracellular fusion events, reconstitution is particularly valuable. Early work on intracellular trafficking used a cell-free assay (Balch et al., 1984) that was critical in identification of key molecular components (Rothman, 2014). Later work used minimalistic components to show that *soluble NSF attachment protein receptor* (SNARE) proteins are sufficient to drive membrane fusion, albeit slowly (Weber et al., 1998) and that SNARE copy numbers determine the size of releasable cargo (Shi et al., 2012; Bello et al., 2016). Although very useful, these bulk studies were limited in the information they could provide. For example, the overall fusion rate is often limited by the docking rate (Smith and Weisshaar, 2011; Xu et al., 2015), so post-docking stages cannot be probed in detail. To overcome these issues and to monitor post-docking stages, assays sensitive to single docking and fusion events were developed both for studying SNARE-mediated mechanisms (Bowen et al., 2004; Fix et al., 2004; Liu et al., 2005; Karatekin et al., 2010; Kiessling et al., 2017) and viral fusion (Floyd et al., 2008; Costello et al., 2013; Bulow et al., 2020). In these assays, a particle mimicking a synaptic vesicle, a small unilamellar vesicle (SUV) reconstituted with neuronal v-SNARE proteins (vSUV), docks and fuses with a planar bilayer supported on a glass substrate, reconstituted with the cognate t-SNAREs (tSBL), as shown in Figure 1A. The vSUV is labeled with fluorescent lipids whose transfer to the supported bilayer (SBL) is monitored. The geometry is ideal for monitoring docking and fusion of the vSUV using total internal reflection fluorescence microscopy (TIRFM), which results in excellent signal-to-noise ratio (Axelrod, 2008). For viral fusion, the vSUV is replaced by a virus (Floyd et al., 2008; Bulow et al., 2020) or a virus-like particle (VLP) (Costello et al., 2013). The supported bilayer can be produced by spreading and fusion of t-SNARE liposomes coated with a poly-ethylene glycol (PEG) cushion (Karatekin et al., 2010; Karatekin and Rothman, 2012), Langmuir-Blodgett deposition of a monolayer followed by fusion with t-SNARE liposomes (Kiessling et al., 2017), or using vesicles derived directly from the plasma membrane of cells expressing a protein of interest (Costello et al., 2013). An alternative to the use of a supported bilayer is to surface-tether target vesicles that bind and fuse with cognate proteoliposomes (Diao et al., 2010; Diao et al., 2013; Kyoung et al., 2013; Lai et al., 2013; Lai et al., 2014). These assays provided detailed mechanistic insights, such as the docking-to-fusion delays reflecting how rapidly t-SNAREs are recruited to a docked vSUV (Karatekin et al., 2010), hemifusion intermediates (Floyd et al., 2008), the number of fusogens needed for efficient fusion (Floyd et al., 2008; Domanska et al., 2010; Karatekin et al., 2010), or role of additional proteins that synchronize fusion to the moment calcium increases (Lai et al., 2017). However, despite their power, these fluorescence-based assays are usually not informative about fusion pore dynamics, because acquisition rates are too slow and/or interpretation of release kinetics is not straightforward.

**FIGURE 2:**
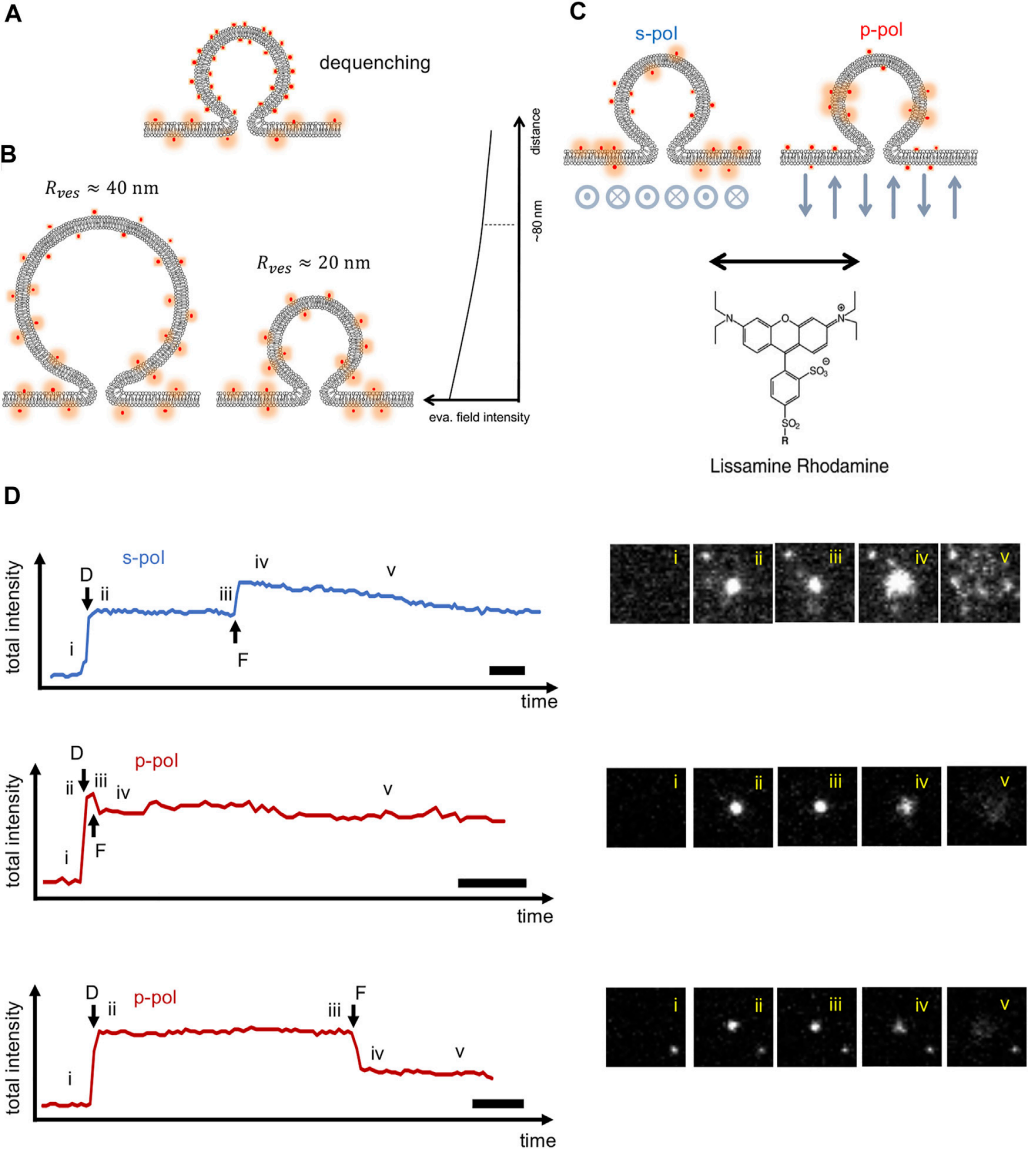
Factors contributing to fluorescence intensity changes observed during SUV-SBL fusion. **(A)** Dequenching. At sufficiently high density in the SUV membrane, lipid labels are self-quenched prior to fusion. Fusion with the SBL allows the fluorophores to disperse and dequench. In our experiments, the fluorophore density is kept to 1 mol %, minimizing dequenching ( ≲10% contribution). **(B)** Evanescent field decay. Fluorophores closer to the glass-water interface experience a stronger excitation field and are brighter, because the evanescent field decays rapidly going away from the interface. Upon fusion, the fluorophores are transferred to the SBL, closer to the interface, so the total intensity increases. The effect is stronger for larger SUVs, since on average the approach to the interface is larger. **(C)** Excitation polarization. A fluorophore with an excitation dipole parallel to the membrane will be excited more efficiently in the SBL than the SUV using s-pol excitation [electric field perpendicular to the plane of incidence, indicated by the symbols below the SBL representing arrows going out of the plane of the drawing toward the viewer (dot in a circle) or the other direction (cross inside circle)]. The opposite is true for p-pol excitation (the polarization direction is indicated by the up-down arrows). Note that for all polarizations other than s-pol, the electrical evanescent field is in fact elliptically polarized [see e.g., Axelrod et al. (1984)], which is not shown for simplicity. The structure of the lissamine rhodamine chromophore is shown (R is a phosphatidyl ethanolamine lipid). On average, the excitation dipole (double arrow) is expected to be nearly parallel to the membrane (Crane et al., 2005). **(D)** Examples of total fluorescence intensity profiles of fusion events recorded with s- or p-pol excitation. LR-PE labeled vSUVs were fused with tSBLs while image stacks were recorded in TIRFM at 56 Hz. Every box is 8 μm -by- 8 μm (30 pixels by 30 pixels), large enough that diffusion of lipids outside the box is negligible during the analysis period. Using s-pol, fusion always results in an increase in total fluorescence **(top)**, because both the evanescent field **(B)** and polarization effects **(C)** contribute positively to the intensity change. Using p-pol excitation, the two effects compete, resulting in a minimal change **(middle)**, decrease **(bottom)**, or increase (not shown). Scale bars represent 200 ms. Snapshots of the events are shown to the right, using the same numbering as in Figure 1.

More direct information about dynamics of single fusion pores using artificial membranes has usually relied on electrical measurements (Melikyan et al., 1993a; Melikyan et al., 1993b; Chanturiya et al., 1997; Wu et al., 2016; Wu et al., 2017; Bao et al., 2018; Dudzinski et al., 2018; Karatekin, 2018; Dudzinski et al., 2019; Das et al., 2020). However, unlike TIRF microscopy based assays, electrical signals are usually not suitable to monitor pre-fusion stages, since a signal appears only after a fusion pore opens. Despite its potential, TIRF microscopy analysis of reconstituted membrane fusion events has typically been limited to extracting rates of docking and fusion, and docking-to-fusion delays for individual vesicles (Floyd et al., 2008; Domanska et al., 2010; Karatekin et al., 2010; Karatekin and Rothman, 2012; Stratton et al., 2016; Kreutzberger et al., 2017). Lipid mixing kinetics are challenging to study quantitatively, because lipid transfer presumably starts with the opening of the initial fusion pore, only ∼1–2 nm wide, but the spread of the signal is not visible until the lipids diffuse a distance on the order of the optical resolution (∼250 nm), which may take ∼10 ms or longer. Spreading kinetics will therefore reflect a convolution of the actual release kinetics through the fusion pore and diffusion of the labels on the SBL surface. For this reason, it is better to rely to on intensity changes as a fluorophore is transferred from the SUV into the SBL (Figure 1B) but there are at least three intertwined factors that may contribute to such an intensity change which complicate analysis (Figure 2): 1) dequenching if the labeling density is too high, 2) evanescent field decay, and 3) polarization effects.

Dequenching always contributes an increase in fluorescence intensity change upon fusion (Figure 2A). However, this contribution is difficult to quantitatively relate to lipid release kinetics, because of the highly non-linear dependence of energy transfer on fluorophore density. Fortunately, this effect can easily be avoided by using dilute labeling. Evanescent field decay similarly always contributes an increase in intensity upon transfer of a label from the SUV into the SBL, because on average, fluorophores move closer toward the glass-water interface where excitation is stronger (Figure 2B). The magnitude of this effect depends on vesicle size, $R_{ves}$ , because the average change in fluorophore distance from the interface is determined by vesicle size. Finally, polarization effects can contribute to an increase or decrease in signal, depending on whether the label used is excited more efficiently while in the SUV or in the SBL (Figures 2C,D). This effect depends on the orientation of the label’s excitation dipole with respect to the membrane and the polarization of the excitation light (Anantharam et al., 2010). Because of the unique properties of evanescent waves, polarization can be nearly purely parallel (p-pol) or perpendicular to the plane of incidence (s-pol), unlike for wide-field microscopy where only s-pol is possible (Axelrod, 2008). Thus, the final change in signal is a complex combination of possibly competing effects. The challenge then is to quantitatively relate fluorescence intensity changes accompanying a membrane fusion event to the kinetics of lipid transfer from the SUV to the SBL. Since the magnitude of the second effect depends on vesicle radius $R_{ves}$ , this quantity needs to be estimated. Knowing $R_{ves}$ is also required to compare the release rate to what is expected from diffusion-limited kinetics. If the fusion pore slows release by flickering or by another mechanism, release would be slower than expected for diffusion.

Fortunately, in the SUV-SBL TIRF microscopy assay the signal-to-noise is good enough to detect single lipid labels such as lissamine rhodamine linked to phosphatidylethanolamine (LR-PE) as the labels spread in the SBL after fusion (Figure 1B). Direct measurement of single lipid label intensity in the SBL, $I_{lip}$ , is key to estimating SUV size: given $I_{lip}$ , vesicle radius $R_{ves}$ is deduced from the known labeling density of the SUV membrane and the total intensity change $\lambda_{TIRF}$ after all labels have been transferred into the SBL. Stratton et al. (2016) estimated $R_{ves}$ for every fusion event, from which they calculated the release time expected for lipid diffusion. In many cases the actual release time was much slower, suggesting a flickering fusion pore hindered release. A “pore openness” parameter $P_{0}$ (the fraction of time the pore is open during a flickering episode) was used to characterize how much the pore slowed release.

Because the reconstitution procedure produces a polydisperse population of SUV sizes and $R_{ves}$ is estimated for every SUV that fused, extrapolation of $\lambda_{TIRF}$ to $R_{ves}\to 0$ can be used to extract a pure polarization contribution to $\lambda_{TIRF}$ (Stratton et al., 2016). However, the effect of the excitation field polarization was not tested by Stratton et al. (2016), as commercial microscopes do not usually allow control of polarization. Here we built a simple TIRF microscope that allows the excitation polarization to be varied continuously, and recorded SUV-SBL fusion events at multiple polarizations. We found that upon membrane fusion, pure polarization effects can lead to an increase or decrease in total fluorescence intensity that can vary by a factor of >2. The optimal polarization is that which results in the maximal change in intensity, facilitating detection efficiency, and estimation of fusion parameters. For fluorophores whose excitation dipole lies nearly parallel to the membrane, such as LR-PE or 1,1′-dioctadecyl-3,3,3′,3′-tetramethylindodicarbocyanine (DiD), s-pol is optimal. A weak correlation between pore openness and vesicle size is noted, illustrating a possible application that is currently not feasible using existing approaches.

## Results

### A Custom Built TIRF Microscope with Polarization Control

Because commercial TIRF microscopes do not allow polarization control, we built a TIRF microscope that allows continuous variation of the excitation polarization (Supplementary Figure S1 and *Materials and Methods*). The light exiting each laser is polarized, with a polarization ratio >1:100 (*Materials and Methods*). The outputs from individual lasers are coupled into polarization maintaining fibers, which are then combined into a single fiber using a wavelength division multiplexer, preserving polarization. This fiber is mounted into an optomechanical cage system with a rotation stage (Supplementary Figure S1). The fiber can be rotated continuously manually to vary the polarization of the excitation beam. The beam is expanded and deflected by a motorized mirror that also sets the position of the beam focused at the back focal plane, thereby controlling the incidence angle. The setup is controlled through the open-source software micro-manager (Edelstein et al., 2014) (see *Materials and Methods*). Supported bilayers were generated using the vesicle fusion method in microfluidic channels, as described in Karatekin and Rothman (2012). Rotating the excitation polarization resulted in a sinusoidal variation of the mean fluorescence intensity of LR-PE doped SBLs as expected (Supplementary Figure S2). A Glan-Taylor prism can be inserted into the rotation mount before the beam expansion optics to improve the polarization ratio, but this makes it more challenging to maintain the laser beam’s position at the back focal plane of the objective (hence the evanescent depth) fixed as the rotation mount is rotated.

For SUV-SBL fusion experiments, the SBL is reconstituted with neuronal/exocytotic t-SNARE proteins Syntaxin-1 and SNAP25. After incubation of the coverslip with SUVs for at least 30–60 min, the microfluidic chamber is rinsed. The formation of a homogenous and continuous SBL is verified using the 1,2-dioleoyl-sn-glycero-3-phosphoethanolamine-N-(7-nitro-2-1,3-benzoxadiazol-4-yl) (NBD-PE) label in the SBL. Fluorescence recovery after photobleaching of NBD-PE was used to ensure the fluidity of the SBLs (*Materials and Methods*). If a SBL passed these quality checks, SUVs reconstituted with the cognate neuronal/exocytic v-SNARE vesicle-associated membrane protein 2 (VAMP2, also known as synaptobrevin-2) were introduced at a continuous flow rate of 2 μL/min (with a flow cell cross-section of 300 μm by 75 μm, the mean linear flow rate was ∼1.5 mm/s). The SUVs were labeled with LR-PE, excited at 561 nm. We monitored vSUV docking and fusion events continuously using TIRF microscopy, and recorded stream acquisitions at 56 frames/s. As control experiments, we incubated the tSBLs with a solution containing the soluble cytoplasmic domain of VAMP2 (CDV) that competes with the full-length VAMP2 on SUVs for binding the t-SNAREs on the SBL. The rate of fusion events (normalized to SUV lipid concentration and detection area) was 5-fold smaller for the control, consistent with previous reports (Karatekin et al., 2010; Nikolaus and Karatekin, 2016; Stratton et al., 2016).

We first reproduced previous results (Stratton et al., 2016) obtained with a commercial TIRF microscope (Nikon Eclipse Ti), in which the excitation polarization was fixed to s-pol (perpendicular to the plane of incidence, Figure 2C, corresponding to θ=0° on our rotation stage). With this polarization, the total intensity in a 22 μm by 22 μm box (82 by 82 pixels) around the docking/fusion site first increases suddenly upon docking of a SUV to a value $I_{dock}$ (Figure 1B). After a delay $\tau_{dock}$ , when the intensity has decreased slightly to a value $I_{fus}$ due to photobleaching in the SUV, membrane fusion results in a second increase in the total intensity over timescale $\tau_{release}$ as the fluorophores are transferred from the SUV into the SBL where they are brighter. This increase tends toward a maximum value $I_{SBL}$ but reaches a value $I_{max}<I_{SBL}$ due to photobleaching. The total intensity is lower by a factor $\lambda_{TIRF}=I_{dock}/I_{SBL}$ in the SUV compared to the SBL. After reaching $I_{max}$ , the intensity decays with characteristic timescale ${\text{τ}}_{bleach}$ due to photobleaching which is stronger for fluorophores in the SBL (the box size is chosen large enough that no labels leave the box during this time).

If the fusion pore did not hinder release, then release kinetics would be limited by how rapidly the fluorophores diffuse around the SUV and occur on a time scale $\tau_{ves}=A_{ves}/D_{lip}$ , where $A_{ves}$ is the SUV area and $D_{lip}$ lipid diffusivity. By contrast, if the fusion pore slowed release, $\tau_{release}$ would be significantly longer than $\tau_{ves}$ . Stratton et al. (2016) defined a pore openness, $P_{o}$ , to quantify the degree by which the fusion pore impedes release kinetics:

$$P_{o}=g\frac{\tau_{ves}}{\tau_{release}},\;\tau_{ves}=A_{ves}/D_{lip}$$ (1)

where $g=b/2\;\pi \;r_{p}$ is a factor that reflects the role of the pore geometry on lipid release rate. With typical values for the height of the pore (Breckenridge and Almers, 1987) b=15 nm and the fully open pore radius $r_{p}=3$ nm, g is of order unity (Stratton et al., 2016). Note that $r_{p}$ includes half the bilayer thickness (2 nm), i.e., the radius of the aqueous lumen of the pore is 1 nm. For a two-state (open-closed) pore, $P_{o}$ is the fraction of the time the pore is in the open state. For a flickering pore with a continuously varying size in time Eq. 1 is equally valid, with $P_{o}$ the time-averaged pore radius relative to the fully open pore radius (Stratton et al., 2016).

Thus, if we knew ${\text{τ}}_{v\text{es}}$ , we could deduce whether the fusion pore significantly impedes release. We can in fact estimate ${\text{τ}}_{v\text{es}}$ for every fusion event, from combining the intensity of the docked vesicle, $I_{dock}$ , single labeled-lipid intensity in the SBL, $I_{lip}$ , the intensity reduction factor $\lambda_{TIRF}$ , the known labeling density $\rho_{lip}$ , and the lipid diffusivity, $D_{lip}$ . As the lipid labels diffuse away from the fusion site, individual labeled lipids become discernible and thus single-lipid intensity in the SBL, $I_{lip}$ (Supplementary Figure S2), and lipid diffusivity, $D_{lip}$ , can be measured from single particle tracking. This intensity is reduced by a factor $\lambda_{TIRF}$ in the SUV, i.e., the intensity of a single lipid label in the SUV, averaged over all locations in the vesicle, is $\lambda_{TIRF}\;I_{lip}$ . Thus,

$$A_{ves}=I_{dock}/\left(\lambda_{TIRF}\;I_{lip}\;2\rho_{lip}\right).$$ (2)

Estimation of $\lambda_{TIRF}$ is not trivial, because of photobleaching. For a good estimate, fitting of the total intensity time profile to a model is needed. However, hindering of release by the fusion pore can qualitatively modify the kinetics of release. Stratton et al. (2016) considered two limiting cases for release kinetics 1) diffusion-limited release (lipids are released as rapidly as they can diffuse through the pore’s neck), 2) pore-limited release (the pore slows release by flickering). These two limiting cases produce qualitatively different release kinetics (Stratton et al., 2016). In the former case, the fraction of labeled lipids remaining in the vesicle a time t after the pore first opens, $\phi_{ves}$ , decays with an inverse time dependence, $\phi_{ves}=\tau_{ves}/t$ , whereas the latter produces an exponential decay, $\phi_{ves}=e^{-t/\tau_{release}}$ . Since a priori it is not known which limiting case describes release better, a procedure was adopted by Stratton et al. (2016) whereby it is assumed release is pore-limited. The total intensity profile $I_{tot}\left(t\right)$ was then fitted to the kinetics expected for this case to extract $\tau_{release}$ and $\lambda_{TIRF}$ . In addition, lipid diffusivity, $D_{lip}$ , was measured by tracking of individual lipid dyes as they became discernible in the SBL (Stratton et al., 2016). Combining these parameters allowed estimation $A_{ves}$ , $\tau_{ves}$ , and $P_{o}$ (Eqs. 1, 2). Only small values of $P_{o}$ are consistent with the pore-limited release assumption. If this procedure produced a $P_{o}$ value nominally ≥1 , the event was flagged as a diffusion-limited release. It was then verified that the intensity profiles for such events are better described by the diffusion-limited release case.

No significant differences were found between results obtained using the custom-built pTIRF set to s-pol excitation or the Nikon Eclipse Ti TIRF microscope (Stratton et al., 2016), validating measurements with the new instrument.

### Improved Analysis of Fluorescence Intensity Changes Accompanying SUV-SBL Fusion Events

One of the major bottlenecks with the procedure above is estimating lipid diffusivity by tracking of individual lipids. In addition, with polarizations that produced lower fluorescence intensities in the SBL that made single-particle tracking even more challenging, the procedure described above was not practical here. We therefore adopted a modified procedure in which the total fluorescence intensities in five concentric circles of increasing size were fitted simultaneously to a model function which captures both the radial shape of the point-spread function and the expected radial spreading of the released dye due to diffusion (Supplementary Figure S3 and *Materials and Methods*). This procedure provided a more constrained fit and allowed us to estimate the lipid diffusion coefficient, $D_{lip}$ , in addition to the bleaching time, $\tau_{bleach}$ , the lipid release time, $\tau_{release}$ , and the intensity reduction factor $\lambda_{TIRF}$ as independent parameters in the fit. The vesicle intensity just after docking, $I_{dock}$ was extracted from the intensity profile with the largest circle radius (11 pixels). The bleaching rate was assumed to be proportional to excitation efficiency (i.e., bleaching in the SUV was assumed to be $\lambda_{TIRF}$ times the rate in the SBL). To estimate the single lipid intensity $I_{lip}\left(\theta \right)$ , we tracked single labeled lipids in the SBL for θ=0° (s-pol) for which single-lipids were brightest (Supplementary Figure S2). For other polarizations, tracking was not feasible or reliable, so we measured the variation of the average SBL intensity, labeled with 0.5% LR-PE, as a function of polarization angle and used this information as correction factor (Supplementary Figure S2). We then used Eq. 2 to calculate the vesicle area $A_{ves},$ and Eq. 1 to obtain the pore openness. A flowchart summarizing the procedure is shown in Supplementary Figure S4. $P_{o}$ quantifies how much lipid release is slowed compared to free diffusion through an un-restricted fusion pore. For our system, we have previously shown that pore flickering is the main mechanism of release-slowing (Stratton et al., 2016), enabling $P_{o}$ to be interpreted as a duty-cycle, but it can equally be used to empirically quantify release-slowing in systems where the mechanism has not been established.

Using this procedure, and for θ=0° , we found that for most events, lipid release was slower than expected for lipid diffusion, i.e., $\tau_{release}\gg \tau_{ves}$ . Pore openness values were $0.0003<P_{o}<0.90$ for most fusion events (78%), with mean = 0.39. For 19% of the pores, the procedure returned a nominal $P_{o}$ value >1 , indicating a “permanently” open pore, $P_{o}=1$ . Note that “permanently” here means that the pore was open long enough that all lipid labels were released during a single flicker. For the largest vesicles studied $\left(R_{ves}\approx 80\;\mathrm{nm}\right)$ , $\tau_{ves}\approx 70\;\mathrm{ms}$ (with $D_{lip}\approx 1.1\;\mu m^{2}/s$ ), so “permanently open” pores could in fact be flickering at frequencies <14 Hz. Overall, these results are consistent with those reported previously (Stratton et al., 2016), validating the new procedure.

### A Pure Polarization Effect Is Isolated by Extrapolating the Size of the Fusing Vesicles to Zero

**FIGURE 3:**
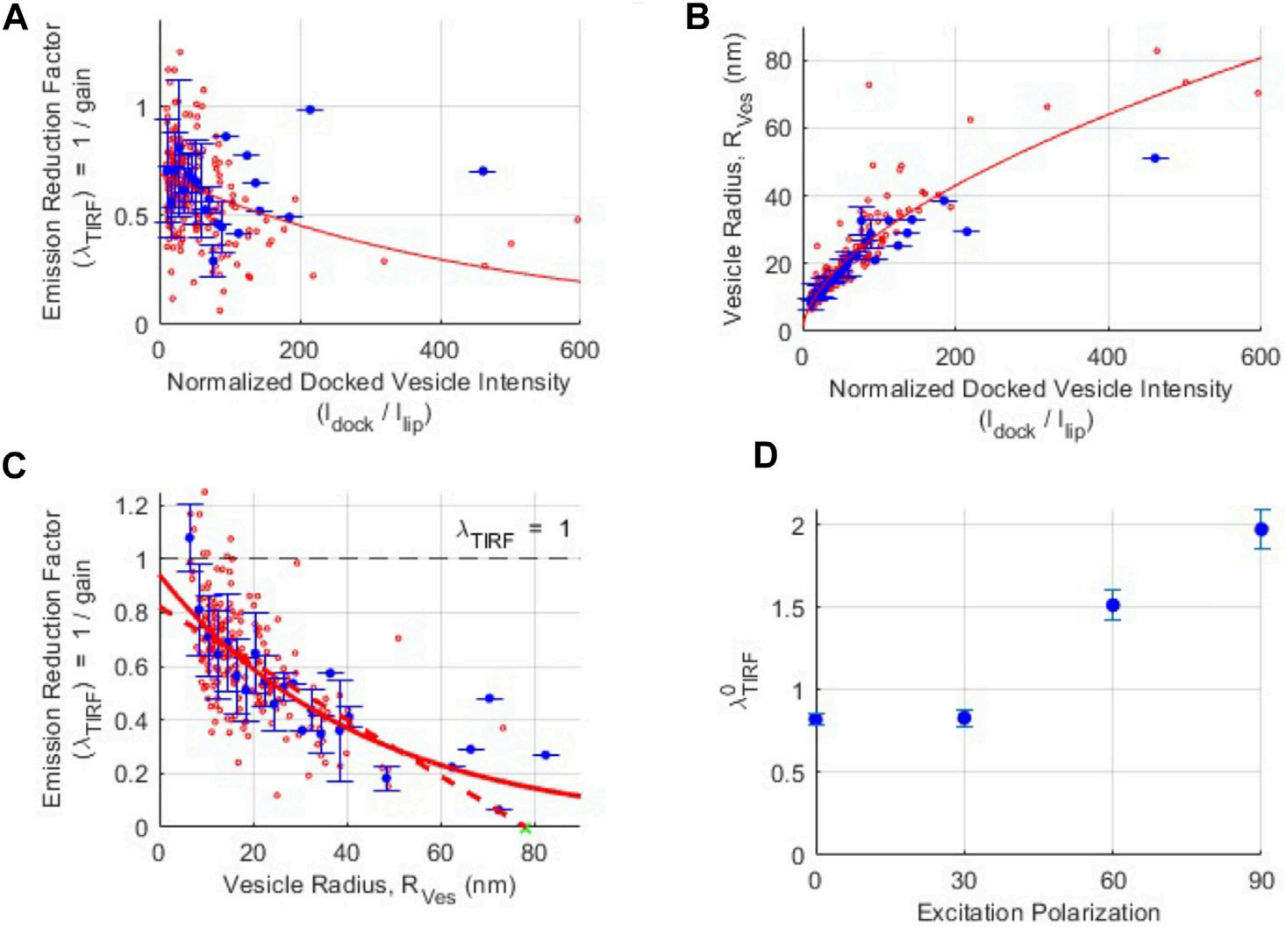
Isolation of the pure polarization contribution to changes in fluorescence intensity during SUV-SBL fusion. **(A)** The relationship between the fluorescence intensity reduction factor $\lambda_{TIRF}$ and docked SUV intensity $I_{dock}$ normalized by single lipid intensity in the SBL, $I_{lip}$ . The red curve is a fit to an exponential, $\lambda_{fit}=a_{1}\;\exp\left(-b_{1}\;I_{dock}/I_{lip}\right)$ , with best-fit parameters (and 95% confidence intervals) $a_{1}=0.69$ (0.65, 0.74), and $b_{1}=2.1\times 10^{-3}$ ( $1.1\times 10^{-3}$ , $3.1\times 10^{-3}$ ). The red dots represent individual events. The blue dots are binned values, with bin size = 2. The error bars represent ± standard deviation (SD). **(B)** Vesicle radius, $R_{ves}$ , as a function of the normalized docked SUV intensity, $I_{dock}/I_{lip}$ . The relationship is fit to a power law, $R_{ves,\;fit}=a_{2}\;{\left(I_{dock}/I_{SBL}\right)}^{b_{2}}$ , with best fit parameters $a_{2}=2.1$ (1.7, 2.4), and $b_{2}=0.57$ (0.54, 0.61). Red and blue dots are as in A, with bin size = 2. **(C)** Fluorescence intensity reduction factor $\lambda_{TIRF}$ as a function of vesicle radius $R_{ves}$ . The continuous red curve is an exponential fit to the data, $a_{3}\;\exp\left(-b_{3}\;R_{ves}\right)$ , with best fit parameters $a_{3}=0.94\;\left(0.87,1.02\right)$ and $b_{3}=0.023\;\left(0.019,0.028\right)$ . The red dashed line is a fit to the small $R_{ves}$ values $\left(R_{ves}\leq 35\;nm\right)$ with the *x*-intercept constrained at the independently estimated evanescent field depth, $\delta_{TIRFM}=78\;\mathrm{nm}$ (see text and *Materials and Methods*). The *y*-intercept of this line, $\lambda_{TIRFM}^{0}=0.82\pm 0.03$ , is our best estimate of the purely polarization contribution to the intensity change as a lipid-dye is transferred from a SUV into the SBL upon fusion. Red and blue dots are as in **(A)**, with bin size = 1 nm. **(D)** Fluorescence intensity reduction factor due to pure polarization effects, $\lambda_{TIRFM}^{0}$ , as a function of excitation polarization angle, θ . Estimates of $\lambda_{TIRFM}^{0}$ at θ=30°, 60° and 90° were done similarly to **(A–C)** for θ=0° , see Supplementary Figure S5. For A-C, 195 events were analyzed, for **(D)**, a total of 446 events were analyzed.

What is the contribution of the excitation polarization to the intensity change $\lambda_{TIRF}$ as a lipid-dye is transferred from the SUV to the SBL membrane? Here we use an extrapolation procedure to estimate this quantity. We start with the relationship between the vesicle area (hence vesicle radius $R_{ves}=\sqrt{A_{ves}/\left(4\pi \right)}$ ), the intensity reduction factor, $\lambda_{TIRF}$ , and the normalized docked intensity, $I_{dock}/I_{lip}$ , through Eq. 2 (note that the labeling density $\rho_{lip}$ is fixed). Thus, the vesicle radius is a function of $\lambda_{TIRF}$ and $I_{dock}/I_{lip}$ . The parameters $\lambda_{TIRF}$ and $I_{dock}/I_{lip}$ are actually not independent from one another, but the theoretical relationship between the two is complex (Stratton et al., 2016). Thus, we determined an empirical relationship between these two quantities by plotting $\lambda_{TIRF}$ as a function of $I_{dock}/I_{lip}$ in Figure 3A for θ=0° (s-pol). Using this relationship, one can obtain vesicle size either as a function of the normalized docked intensity, $R_{ves}\left(I_{dock}/I_{lip}\right)$ , or as a function of the intensity reduction factor, $R_{ves}\left(\lambda_{TIRF}\right)$ . Both representations are useful. The former is plotted in Figure 3B, which shows that the normalized docked intensity uniquely determines $R_{ves}$ . That this should be case is not entirely obvious, because the polarization contribution to $I_{dock}/I_{lip}$ varies as a function of distance from the interface in a non-monotonic manner (Stratton et al., 2016). The latter representation is inverted, then the value of $\lambda_{TIRF}$ as $R_{ves}\to 0$ is extrapolated to estimate the pure polarization contribution, as the evanescent field decay contribution vanishes at this limit. It was shown by Stratton et al. (2016) that the slope at the origin is ${\text{λ}}_{TIRF}^{0}/{\text{δ}}_{TIRFM}$ , where $\lambda_{TIRF}^{0}$ is the value of the intensity reduction factor extrapolated to zero vesicle radius and $\delta_{TIRFM}$ is the characteristic decay length of the evanescent field. For the incidence angle used, we independently estimated $\delta_{TIRFM}\approx 78\;\mathrm{nm}$ here (*Materials and Methods*). To estimate the slope at origin, we fitted a line to $R_{ves}\leq 35\;\mathrm{nm}$ values, while constraining the *x*-intercept to be equal to $\delta_{TIRFM}\approx 78\;\mathrm{nm}$ , obtaining, slope=−0.0105±0.0004 nm , and ${\text{λ}}_{TIRF}^{0}=0.82\;$ (95% confidence interval, CI=0.79−0.85 ).

We repeated the procedure described above at additional excitation polarizations (Supplementary Figure S5), finding $\lambda_{TIRFM}^{0}=0.83\;\left(CI=0.78-0.88\right)$ , 1.51 (1.43−1.60) , and 1.97 (1.85−2.01) for θ=30 , 60 , and 90° , respectively (Figure 3D). Thus, for θ = 60° and 90° (p-pol) cases, the pure polarization contribution is a decrease in total intensity upon lipid transfer from the SUV to the SBL, with $\lambda_{TIRFM}^{0}>1$ . At finite values of $R_{v\text{es}}$ , the polarization effect competes with the intensity enhancement from the evanescent field decay effect (Figure 2). The two contributions are equal at $R_{ves}^{*}=22.6\;\text{nm}\;\left(\text{CI}=20.0-25.8\;\text{nm}\right)\text{ and }36.8\;\text{nm }\left(30.4-47.9\;\text{nm}\right)$ for θ=60° and 90° , respectively (Supplementary Figure S5). For these polarizations, 59, and 80% of all events, respectively, had $\lambda_{TIRF}>1$ . By contrast, only 4% of all events for θ=0° or 30° had $\lambda_{TIRF}>1$ , due to noise. For vesicle sizes close to $R_{ves}^{*}$ , the net change in the total intensity upon fusion is near zero, such as the case depicted in Figure 2D, middle. For such cases, analysis and extraction of a $P_{o}$ value is particularly challenging.

Overall, these results show that for polarizations that contribute a decrease to the change in total intensity upon SUV-SBL fusion $\left(\lambda_{TIRFM}^{0}>1\right)$ , there is a polarization-dependent vesicle size for which the net intensity change is nil, making analysis of lipid mixing kinetics very challenging. For the commonly used lipid dye LR-PE, pure polarization effects can vary by a factor of ∼2.4; importantly, a polarization with a net positive contribution to the signal change can be chosen to ensure all events can be analyzed for lipid mixing kinetics.

### The Effect of Excitation Polarization on Release Parameter Estimates

Two of the main bottlenecks in the analysis pipeline are visual identification of fusion events and single-molecule tracking of lipids for estimation of $D_{lip}$ (Supplementary Figure S4). When using non-optimal excitation polarizations, the most obvious effect is that it becomes much harder to visually identify fusion events and distinguish them from undocking events. Thus, considerably more time is spent on identification of events. Although we did not use an automated event-detection algorithm, it is likely that event detection would be more challenging for such an algorithm for non-optimally excited samples.

Once fusion events are correctly identified, with non-optimal excitation polarizations the most important challenge for analysis is a loss of sensitivity to single lipids diffusing in the supported bilayer after vesicle fusion. This made single-molecule tracking based estimation of lipid diffusion more challenging in all but the s-polarized case. We were able, however, to estimate diffusion coefficients based on the lateral spread of the released dye, with these estimates being broadly compatible across polarizations. The distribution of other fitted parameters was also broadly similar across all excitation polarizations (e.g., see distributions for ${\text{τ}}_{dock}$ ; Supplementary Figure S6). We nonetheless observed subtle differences in the observed distribution of pore openness $\left(P_{o}\right)$ , with an apparent reduction in the number of slow-release events (small $P_{o}$ values) for p-polarization (Supplementary Figure S6). A potential explanation for this is that the relative fluorescence enhancement/reduction is much less pronounced than for p polarized case, making it significantly harder to unambiguously estimate the time at which the fusion pore opens. This is particularly likely for the slower release events where, in the absence of an enhancement on fusion, the docked vesicle signal will continue to dominate until well after the fusion pore opens.

Together these results let us conclude that choosing an optimal excitation polarization (e.g., s-polarized for LR-PE) is desirable, but that with knowledge of the actual polarization state and a suitable numeric model, useful measurements can be obtained on systems (such as many off-the shelf commercial TIRF microscopes) where polarization may not be controllable nor optimal.

### Fusion Pore Openness, the Number of v-SNAREs per Liposome, and Membrane Curvature

As an illustrative application of pTIRF microscopy to the study of fusion pores, we explored the relationship between fusion pore openness and SNARE copy numbers and membrane curvature. How membrane fusion depends on SNARE copy numbers has been studied both using bulk and single-event assays. In single-event SUV-SBL fusion assays, it was found that 5–10 *trans*-SNARE complexes (SNAREpins) are required for rapid membrane fusion (Domanska et al., 2010; Karatekin et al., 2010). Bulk SUV-SUV fusion studies reported as few as a single SNAREpin could mediate lipid mixing (van den Bogaart et al., 2010), but content release required more (Shi et al., 2012), suggesting more SNAREpins drive larger fusion pores. Consistent with this idea, monitoring release of differently sized cargo in a nanodisc-SUV bulk fusion assay, Bello et al. showed that release of larger cargo required more SNAREpins (Bello et al., 2016). In nanodisc-based assays where single-pore conductance reflects pore size, it was shown that the mean fusion pore size increases with increasing SNARE copy numbers (Wu et al., 2017; Bao et al., 2018), an effect attributed to entropic repulsion among the SNARE complexes lining the pore’s waist (Wu et al., 2017). These results in reconstituted assays are consistent with observations in live cells that cargo release is faster when more SNAREs are available (Zhao et al., 2013; Acuna et al., 2014; Bao et al., 2018).

**FIGURE 4:**
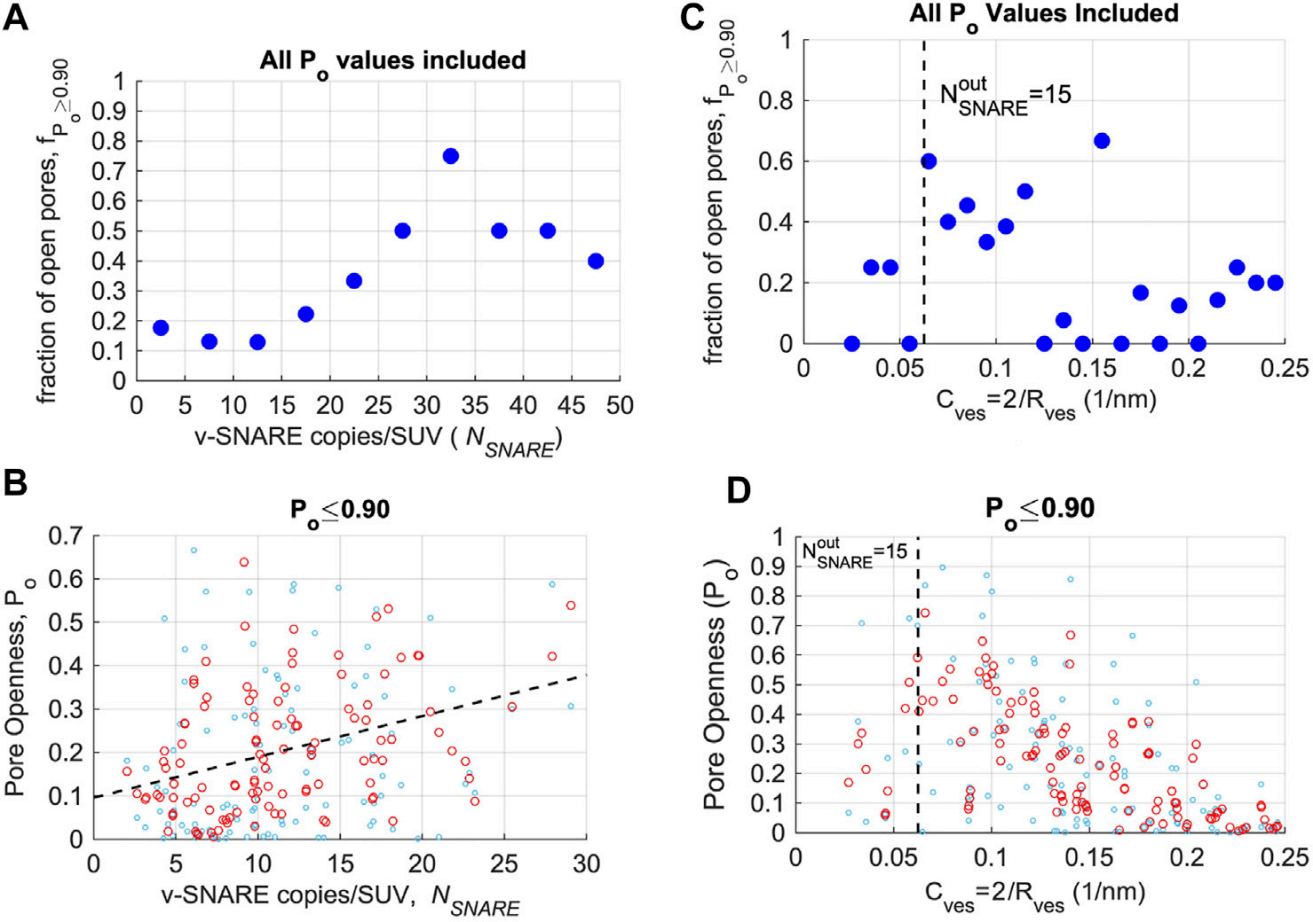
Pore openness as a function of SNARE copies per liposome or vesicle curvature. **(A)** The fraction $f_{P_{o}\geq 0.90}$ of open pores $\left(P_{o}\geq 0.90\right)$ , as a function of average v-SNARE copies per SUV, $N_{SNARE}$ . The fraction increases up to $N_{SNARE}\approx 30$ , to reach ∼0.5. **(B)** Pore openness for flickering pores $\left(P_{o}<0.90\right)$ , as a function of $N_{SNARE}$ . The red circles are original data points (light blue) smoothed with a moving average filter with span = 5. The dashed line is a linear fit to the smoothed data with slope 0.0094 (95% confidence interval = 0.0051–0.0138, $R^{2}=0.15$ ). Note that because v-SNAREs are reconstituted with random orientation, only ∼1/2 would face outside and contribute to fusion. **(C)** There is no clear correlation between the fraction $f_{P_{o}\geq 0.90}$ of open pores $\left(P_{o}\geq 0.90\right)$ and total membrane curvature, $C=2/R_{ves}$ . **(D)** Pore openness for flickering pores ( $P_{o}<0.90$ ), as a function of membrane curvature. For flickering pores $\left(P_{o}<0.90\right)$ , $P_{o}$ decreases with increasing curvature for vesicle curvatures that correspond to $N_{SNARE}^{out}\approx 15$ (indicated by a vertical dashed line) or smaller. Symbols represent original and smoothed data points as in **(B)**. For **(A,C)**, 164 events were analyzed. For **(B,D)**, 128 events were analyzed.

To test if pore openness, $P_{o}$ , depended on the number of v-SNAREs per liposome, $N_{SNARE}$ , we plotted the fraction of open pores $\left(P_{o}\geq 0.9\right)$ as a function of $N_{SNARE}$ (Figure 4A). Because our $P_{o}$ estimates are most reliable for θ=0° , we used the corresponding data set for this analysis. The fraction of open pores $f_{P_{o}\geq 0.90}$ increased with $N_{SNARE}$ up to $N_{SNARE}\approx 30$ , above which it plateaued around 0.5. We explored this relationship further, by plotting $P_{o}$ values for flickering pores $\left(P_{o}<0.9\right)$ as a function of $N_{SNARE}$ . There was a weak trend for $P_{o}$ to increase with increasing $N_{SNARE}$ , up to $N_{SNARE}\approx 30$ (Figure 4B). Note that because the v-SNAREs are incorporated with a random orientation into the SUVs (Karatekin et al., 2010), the effective number of v-SNAREs facing outside the SUV on these plots, $N_{SNARE}^{out}$ , is ∼1/2 the plotted (total) value.

Membrane curvature could also contribute to fusion pore dynamics. Smaller liposomes fuse faster both in bulk experiments (Malinin et al., 2002; Hernandez et al., 2012; Yang et al., 2021) and computer simulations (Gao et al., 2008), although the effects on fusion pore dynamics are not clear. In PC12 and chromaffin cells, larger vesicles have more stable initial fusion pores (Zhang and Jackson, 2010), suggesting high membrane curvature may contribute to fusion pore expansion. To test for membrane curvature effects, we first plotted the fraction $f_{P_{o}\geq 0.90}$ of open pores $\left(P_{o}\geq 0.9\right)$ as a function of vesicle curvature, $C=2/R_{ves}$ , but no clear correlation emerged (Figure 4C). However, a weak trend emerged for $P_{o}$ values for flickering pores ( $P_{o}<0.9$ ) to decrease as a function of increasing curvature, $C=2/R_{ves}$ , at high curvatures corresponding to the range of liposome sizes for which $N_{SNARE}^{out}≲15$ (Figure 4D).

Overall, these observations suggest that pore openness increases with vesicle area, likely with contributions from both SNARE copy numbers and membrane curvature.

## Discussion

Polarization effects in TIRF microscopy have been exploited in the past to study membrane fusion events both for artificial systems (Kiessling et al., 2010; Stratton et al., 2016) and live secretory cells (Anantharam et al., 2010; Anantharam et al., 2012). For a fixed geometry, the effect of excitation polarization on the observed fluorescence intensity can be predicted with reasonable assumptions (Anantharam et al., 2010). The problem is that the geometry, how it evolves during membrane fusion, and diffusion kinetics of labels are usually not known. Consequently, one usually makes assumptions about vesicle size, fusion pathway, and label diffusion in order to solve the equations describing the polarization-dependent fluorescence intensity along the assumed fusion pathway. For example, Anantharam et al. (2010) showed that for a lipid-dye such as DiI with the excitation dipole oriented nearly parallel to the membrane, and assuming a fixed membrane geometry and homogenous label distribution, a particular combination of p- and s-pol excitation leads to a signal that is proportional essentially only to label density convolved with the distance to the glass-water interface, whereas another combination is sensitive mostly to membrane orientation. They showed that fused secretory granules in chromaffin cells typically retain their shapes for many seconds, consistent with previous reports (Taraska et al., 2003; Tran et al., 2007; Anantharam et al., 2010; Anantharam et al., 2012; Chiang et al., 2014; Karatekin, 2018; Shin et al., 2018), presumably long enough for the DiI label to equilibrate. The approach requires fluorescence signals collected under alternating p- and s-pol excitation and is not suited to monitor the kinetics of rapid lipid redistribution, which is our main interest here. Kiessling et al. (2010) took a similar approach to calculate fluorescence intensity changes observed during SUV-SBL fusion. They labeled SUV membranes with lipid dyes and imaged fusion events either with s- or p-pol excitation (without switching), and collected fluorescence from a small region of interest (ROI). However, interpretation relied on various assumptions, including SUV size. Importantly, they assumed lipids were transferred into the SBL from a SUV because the SUV rapidly (within ∼8 ms) flattened into the SBL. Because simultaneous monitoring of content release and lipid mixing shows that most fusion pores reseal after partial content release in a similar assay (Stratton et al., 2016), the intensity changes observed by Kiessling et al. (2010) more likely represent diffusion of the lipid labels from the SUV into the SBL with the SUV retaining an omega-shape until the lipids are dispersed into the SBL. This would be an interpretation consistent with observations of secretory granule exocytosis in neuroendocrine cells (Taraska et al., 2003; Tran et al., 2007; Anantharam et al., 2010; Anantharam et al., 2012; Chiang et al., 2014; Karatekin, 2018; Shin et al., 2018), enveloped viral fusion events (Melikyan et al., 1993a; Melikyan et al., 1993b; Melikyan et al., 1995; Cohen and Melikyan, 2004), and other reconstitutions with artificial membranes with sufficient resolution to monitor fusion pore dynamics (Chanturiya et al., 1997; Lai et al., 2013; Wu et al., 2016; Wu et al., 2017; Wu et al., 2021), where pore flickering and slow changes in the Ω -shape of the vesicle appear to be the norm.

Using pTIRF microscopy, we previously reported a quantitative analysis of lipid mixing kinetics from fluorescence intensity changes observed during SUV-SBL fusion (Stratton et al., 2016). A key advance was our ability to detect single lipid labels, which allowed estimation of the SUV size for every fusion event. With the SUV area known, release kinetics could be interpreted and related to fusion pore properties quantitatively using a model. However, the approach suffered from two major bottlenecks: visual detection of SUV-SBL fusion events and tracking single lipids to estimate $D_{lip}$ . Here we have improved both of these processes, by optimizing the excitation polarization to make event detection easier and using a modified model for fitting fluorescence profiles so that $D_{lip}$ can be estimated without need for lengthy single particle tracking.

Choosing the optimal excitation polarization is critical, because polarization effects can contribute an increase or a decrease to the total intensity change upon fusion. In the latter case polarization-related intensity variations work against evanescent field decay (and dequenching) effects so the change in total intensity upon fusion is reduced or even abolished. Such events become particularly challenging, if not impossible, to detect and analyze. Excitation light used in commercial TIRF microscopes is typically polarized, but the polarization characteristics are rarely specified and the polarization angle cannot be controlled. Additionally, there seems to be no convention in design, as two commercial systems we tested in the past used s-pol (Nikon Eclipse Ti) or p-pol (Olympus CellTIRF) excitation.

With no possibility of changing the excitation polarization with a commercial TIRF microscope, one could in principle use a fluorophore with the excitation dipole oriented with respect to the membrane such that membrane fusion results in a maximal increase in total intensity. However, the orientation of the excitation dipole moment for many fluorophores is not known and compromises may need to be made in other parameters such as excitation and emission maxima, photostability, and/or brightness. To overcome these limitations, here we built a TIRF microscope with continuously variable excitation polarization using off-the-shelf components and controlled it using open-source software. We monitored SUV-SBL fusion events using various excitation polarization angles θ . We found that for the commonly used lipid label LR-PE, purely polarization effects can vary >2-fold. The polarization that results in the largest intensity increase upon fusion is optimal for detection of fusion events, greatly reducing the time spent in event-identification.

In addition to optimizing the excitation polarization, we improved the analysis pipeline by a more robust procedure that allows estimation of lipid diffusivity $D_{lip}$ without need for time-consuming single-particle tracking. For initial experiments, the mean intensity of single lipid-linked fluorophores is still required to estimate $R_{ves}$ , but long and continuous tracks are not needed as they would be for estimation of $D_{lip}$ from single particle tracks. Once the relationship between the fluorescence intensity reduction factor ${\text{λ}}_{TIRF}$ and the vesicle radius $R_{ves}$ is determined for a given optical setup, lipid label, and excitation polarization (Figure 3C), subsequent experiments become simpler as the mean single lipid intensity $I_{lip}$ is no longer needed to estimate $R_{ves}$ . Another major advantage of such a calibration is that constraints on frame rates and high intensity illumination can be relaxed. For single lipid detection, high intensity illumination and ∼10–30 ms exposure are typically needed. Shorter exposure times result in poor signal-to-noise, while longer exposures result in motion-blur. With no need for single-lipid sensitivity, acquisition rates can be increased several fold to capture lipid release kinetics with better time-resolution. Although our camera is limited to a sampling rate of ∼56 frames/s full-frame, with cropping and binning, sampling rates of ∼1 KHz or even higher are feasible (note that high spatial resolution is not a critical requirement for this application).

As an illustrative example, we explored how fusion pore dynamics depend on SNARE copy numbers and membrane curvature. We first confirmed our previous finding that lipid mixing kinetics are slower than expected for diffusion for most fusion events, suggesting the fusion pore hinders lipid release (Stratton et al., 2016). In addition, here we have uncovered a weak correlation between pore openness, $P_{o}$ , and the vesicle area, $A_{ves}$ . Both the number of v-SNAREs per liposome, $N_{SNARE}$ , and vesicle curvature C appear to contribute to the trend. The finding that pore openness, $P_{o}$ , increases with $N_{SNARE}$ for small values of $N_{SNARE}$ is consistent with previous findings that increased SNARE copy numbers lead to larger fusion pores (Wu et al., 2017; Bao et al., 2018).

We expect the description of our simple polarized TIRF setup and analysis procedure will be of interest to researchers studying mechanisms of membrane fusion using reconstituted liposomes (Kiessling et al., 2010; Stratton et al., 2016), purified secretory granules (Kreutzberger et al., 2017), synaptic vesicles (Kreutzberger et al., 2019), enveloped viruses (Floyd et al., 2008; Ivanovic et al., 2013; Bulow et al., 2020), virus like particles (Costello et al., 2013), live cell exocytosis (Anantharam et al., 2010; Anantharam et al., 2012), or any other system in which membrane fusion can be monitored using polarized TIRFM.

## Materials and Methods

### Materials

1-palmitoyl-2-oleoyl-sn-glycero-3-phosphocholine (POPC), 1,2-dioleoyl-sn-glycero-3-phospho-L-serine (sodium salt) (DOPS), 1-palmitoyl-2-oleoyl-sn-glycero-3-phosphoethanolamine (POPE), 1-stearoyl-2-arachidonoyl-sn-glycero-3-phosphoethanolamine (SAPE), 1,2-dioleoyl-sn-glycero-3-phosphoethanolamine-N-(lissamine rhodamine B sulfonyl) (LR-PE), 1,2-dioleoyl-sn-glycero-3-phosphoethanolamine-N-(7-nitro-2-1,3-benzoxadiazol-4-yl) (NBD-PE), and 1,2-dioleoyl-sn-glycero-3-phosphoethanolamine-N-[methoxy(polyethylene glycol)-2000] (PEG2K-PE) were purchased from Avanti Polar Lipids (Alabaster, AL). NBD-PE (0.5 mol%) was included only in the t-SNARE SBL to test SBL fluidity using fluorescence recovery after photobleaching (Karatekin and Rothman, 2012; Nikolaus and Karatekin, 2016).

### Recombinant Protein Expression and Purification

Recombinant proteins vesicle-associated membrane protein 2 (VAMP2, also known as synaptobrevin-2), syntaxin-1, and synaptosomal-associated protein 25 (SNAP25) were expressed, purified, and reconstituted into SUVs as described in detail previously (Karatekin et al., 2010; Karatekin and Rothman, 2012). Plasmids were a generous gift from J. E. Rothman (Yale University). A lipid-to-protein ratio (L:P) of 200 was used for vSUVs and 5,000 or 10,000 for tSBLs.

### Preparation of SUVs and SBLs

For reconstitution of protein, we used the method of refs. Karatekin et al. (2010); Karatekin and Rothman (2012). We used the following molar ratios for the vSUVs: POPC/DOPS/(SAPE or POPE)/PEG2KPE/LR = 54/20/20/5/1. The t-SBLs harbored one t-SNARE complex for every 5,000 or 10,000 lipids, and the lipid composition was (in molar ratios) POPC/DOPS/(SAPE or POPE)/PEG2KPE/NBD-PE = 69.5/10/15/5/0.5. As negative controls, we included the soluble cytoplasmic domain of the v-SNARE VAMP2 (CDV, residues 1–92, 10 μM) which associates with the t-SNAREs on the tSBL and inhibits docking and fusion of vSUVs. SUV diameters were 20–200 nm, estimated from dynamic light scattering (DynaPro NanoStar, Wyatt Technology, Santa Barbara, CA, United Sates).

### Estimation of SNARE Copy Numbers Per SUV

For every batch of vSUVs, we calculated the actual lipid-to-protein ratio (LP) following ref. Karatekin et al. (2010). The lipid concentration was estimated using LR-PE fluorescence which was independently calibrated using standard solutions. The protein concentration was estimated from SDS-PAGE gels stained with Sypro Orange (MilliporeSigma, St. Louis, MO) running against a known concentration. Assuming an area per lipid (Hung et al., 2007) $a_{lip}=0.70\;nm^{2}$ we estimated the SNARE density ${\text{Γ}}_{SNARE}=1/\left(LP\times a_{lip}\right)$ for each batch. For most batches, the resulting snare density was $3600\;copies/\mu m^{2}$ , with actual LP≈400 . For every event that could be analyzed, the vesicle area $A_{ves}$ was calculated using Eq. 2, which allowed us to estimate the number of v-SNAREs per SUV, $N_{SNARE}=A_{ves}\times \Gamma_{SNARE}$ .

### Polarized TIRF Microscopy Setup

Outputs from a LuxX 488 nm or 638 nm continuous-wave diode-pumped laser (200 or 150 mW maximum power, respectively, Omicron, Rodgau-Dudenhofen, Germany) were each coupled to a polarization maintaining fiber. The output of a 561 nm laser (150 mW maximum power, Cobolt 04-01 Series, Jive, Solna, Sweden) was modulated by an acousto-optical modulator (PCAOM V-50, Crystal Technology, Inc., Palo Alto, CA) before coupling into another polarization maintaining fiber. The fibers were combined into a single fiber using a polarization maintaining wavelength division multiplexer (OZ Optics, Ottawa, Canada). The fiber carrying the combined wavelengths was mounted onto a manual rotation mount (Thorlabs, Newton, NJ). We set the desired excitation field polarization by rotating this mount. If desired, the polarization ratio can be improved by inserting a Glan-Taylor prism, but in practice this complicates alignment as polarization is rotated and was not used. The beam was then expanded and passed through an adjustable diaphragm before being reflected by a mirror whose position was controlled by a motorized actuator (CONEX-TRB12CC DC servo actuator, Newport, Irvine, CA). The beam then went through a tube lens, an excitation filter (ZET488/10x, ZET561/10x, or ZET640/20x, Chroma), and a dichroic mirror (ZT488rdc, ZT640rdc, Chroma) before focusing onto the back focal plane of an Olympus PlanApo 60x/1.45 Oil TIRF objective, mounted on an inverted microscope (IX81, Olympus, Tokyo, Japan). Fluorescence was collected through the same objective, passed through a HHQ500LP and ET525/50m, HHQ575LP, and ET610/60M, or HQ660LP and ET700/75 (Chroma), and detected using an EM-CCD camera (Ixon-ultra-897, Andor, Belfast, United Kingdom). One pixel corresponded to 265 nm in the sample plane. We stream-recorded 60 s movies (3,300 frames) with exposure time 17.8 ms (duty cycle 18.3 ms). The microscope, including the mirror position for setting the evanescence depth was controlled by micro-manager (Edelstein et al., 2014) (the configuration file is available upon request). All experiments were carried at 32°C, using a heated stage insert (Thermo Plate, Tokai Hit, Shizuoka-ken, Japan).

### Evanescent Field Depth Calibration

The evanescent field depth was estimated by measuring the angle of incidence, θ , of the excitation beam with respect to the normal of the imaging plane, and using (Axelrod, 2008) $\delta_{TIRF\;}=\;\;\lambda_{0}/4\pi {\left(n_{g}^{2}\;sin^{2}\;\theta -n_{w}^{2}\right)}^{-1/2}$ , where $\lambda_{0}=561\;\mathrm{nm}$ is the laser excitation wavelength, and $n_{g}=1.52$ and $n_{w}=1.33$ are the refractive indices for glass and water, respectively. An N-BK7 right-angle prism (20 mm per side, PS908, Thorlabs) was coupled to the TIRF objective using a cover slip and oil matching the refractive index of the glass. Adjusted to the same mirror position used in the SUV-SBL fusion experiments, the laser beam passed from the objective into the prism undeflected but was refracted at the glass-air interface as it emerged from the prism. The beam was projected onto a wall and the simple geometry was used to calculate the angle of incidence, θ=71.8−72.6° , corresponding to $\delta_{TIRF}=77-79\;\mathrm{nm}$ . The highest intensity of the projected spot on the wall was found at 72.2° , $\delta_{TIRF}=78\;\mathrm{nm}$ .

### Microfluidic Channels and SBL Formation

We followed ref. Karatekin and Rothman (2012). Briefly, microfluidic channels were made by bonding a block of poly(dimethyl siloxane) (PDMS) replica of a microfabricated structure onto a glass coverslip. Prior to bonding, holes were punched into the PDMS block using a hole puncher (Schmidt Manual Press, Schmidt Technology, Cranberry Twp., PA) to connect tubing for introducing solutions. Coverslips (24 mm by 60 mm) were treated with air plasma for 10 min in a plasma cleaner (LTD Model SP100 Plasma system, Anatech, United Sates, Sparks, NV) before bonding to the PDMS. The PDMS was not plasma treated but was placed under vacuum for at least 20 min to avoid bubble formation during experiments. After assembly of microfluidic channels, a diluted and degassed tSUV or pfSUV suspension was introduced into the channels and incubated for at least 30 min. Unbound SUVs were rinsed away. SBL fluidity was tested using fluorescence recovery after photobleaching (FRAP) using the 488 nm laser to excite NBD-PE (Karatekin and Rothman, 2012; Nikolaus and Karatekin, 2016). Occasionally, SUVs adhered to the glass coverslip but did not burst and form a continuous fluid bilayer. In such cases, the coverslips were additionally incubated with reconstitution buffer (25 mM HEPES-KOH, 140 mM KCl, 100 µM EGTA, and 1 mM DTT, pH 7.4) with 10 mM Mg^2+^ for at least 30 min and then thoroughly rinsed with Mg^2+^ free buffer.

### Detection and Analysis of Fusion Events

Prior to flowing SUVs into a microfluidic channel, the SBL in the viewfield was continuously bleached by 561 nm excitation to reduce background fluorescence. When the first v-SUVs reached the viewfield, image acquisition was initiated to record a 1-min movie consisting of 3,300 frames. Data was recorded for four different excitation polarizations, 0° (s-pol), 30°, 60°, and 90° (p-pol).

Analysis of vesicle fusion was done offline and began with visual identification of fusion events which were then tracked using the SpeckleTrackerJ plugin (Smith et al., 2011) of ImageJ (Schneider et al., 2012) with subpixel resolution. Tracks started the first frame in which a SUV docked onto the SBL until the frame in which it fused with the SBL, as evidenced by the onset of a sudden change in fluorescence intensity, accompanied by the spread of the signal. The track length defined the docking-to-fusion delay $\tau_{dock}$ . Fusion events were further analyzed using PYME (www.python-microscopy.org) and the Python Anaconda platform. The pixel intensities surrounding a particle’s centroid position were summed within concentric circular regions of interest (ROI) with five different radii (3, 5, 7, 9, and 11 pixels radius). The analysis extended from 10 frames prior to docking until 50 frames after fusion. For each fusion event, the total intensity for all radii were simultaneously fitted to a model function which captures both the radial shape of the point-spread function and the expected radial spreading of the released dye due to diffusion. The use of a model encoding this radial information and fitted to multiple different sized ROIs allowed us to estimate the lipid diffusion coefficient, $D_{lip}$ , the bleaching time, $\tau_{bleach}$ , and the lipid release time, $\tau_{release}$ , as independent parameters in the fit. Our specific model function describing the total intensity within a radius R was as follows:

$$I\left(R,t\right)=\;I_{docked}\left(R,\;t\right)+\;I_{release}\left(R,\;t\right)$$ (3)

where:

$$\begin{array}{l} I_{docked}\left(R,t\right)=e^{\frac{-\left(t-t_{dock}\right)}{\tau_{bleach}}}\int_{0}^{R}PSF\left(r\right)\;dr\{\begin{array}{cc} 0 & \;t<t_{dock}\\ 1 & \;t_{dock}\leq t<t_{fusion}\\ e^{\frac{-\left(t-t_{fusion}\right)}{\tau_{release}}} & t_{fusion}\leq t \end{array}\\ I_{release}\left(R,t\right)=\;Ge^{\frac{-G\left(t-t_{fusion}\right)}{\tau_{bleach}}}\int_{0}^{R}\left[PSF⊗H_{diffusion}\right]\left(r\right)\;dr\{\begin{array}{cc} 0 & t<t_{fusion}\\ \left[1-e^{\frac{-\left(t-t_{fusion}\right)}{\tau_{release}}}\right] & t_{fusion}\leq t \end{array} \end{array}$$ (4)

and PSF is an approximation to the microscope point spread function, $H_{diffusion}=e^{\frac{-r^{2}}{4\pi D_{lip}t}}$ is the 2D diffusion Green’s function, ⊗ represents convolution and $G=1/{\text{λ}}_{\text{TIRF}}$ is the gain in intensity that a dye molecule experiences transiting from the vesicle into the bilayer. The bleaching rate is assumed to be proportional to excitation efficiency. The traces were background subtracted and normalized to the total intensity of the docked vesicle before fitted using a weighted least squares fit. Free parameters were $G,D_{lip},\tau_{release},\tau_{bleach}$ . Fit quality was evaluated visually for every fit and poor fits were excluded from further analysis. Fusion events with docking-to-fusion delays $t_{dock}<4$ frames were excluded, as shorter docking times did not result in good fits.

## Data Availability

All data generated or analysed during this study are included in this published article (and its supplementary material files). In particular, all data underlying the results are provided as matlab.fig files (see Supplementary Materials) from which the plotted data points can be extracted. The algorithm used to fit the fusion traces is available as part of the open-source python-microscopy environment (www.python-microscopy.org), with the majority of the relevant logic found in the “fusionRadial.py” module - https://github.com/python-microscopy/python-microscopy/blob/master/PYME/experimental/fusionRadial.py. A python-microscopy “recipe” for automated analysis, and instructions for its use are available on request.

## References

[B1] Acuna C. Guo Q. Burré J. Sharma M. Sun J. Südhof T. C. (2014). Microsecond Dissection of Neurotransmitter Release: SNARE-Complex Assembly Dictates Speed and Ca2+ Sensitivity. Neuron 82, 1088–1100. 10.1016/j.neuron.2014.04.020 24908488 PMC4109412

[B2] Alabi A. A. Tsien R. W. (2013). Perspectives on Kiss-And-Run: Role in Exocytosis, Endocytosis, and Neurotransmission. Annu. Rev. Physiol. 75, 393–422. 10.1146/annurev-physiol-020911-153305 23245563

[B3] Anantharam A. Onoa B. Edwards R. H. Holz R. W. Axelrod D. (2010). Localized Topological Changes of the Plasma Membrane upon Exocytosis Visualized by Polarized TIRFM. J. Cel Biol. 188, 415–428. 10.1083/jcb.200908010 PMC2819686 20142424

[B4] Anantharam A. Axelrod D. Holz R. W. (2012). Real-time Imaging of Plasma Membrane Deformations Reveals Pre-fusion Membrane Curvature Changes and a Role for Dynamin in the Regulation of Fusion Pore Expansion. J. Neurochem. 122, 661–671. 10.1111/j.1471-4159.2012.07816.x 22671293 PMC3408088

[B5] Axelrod D. Burghardt T. P. Thompson N. L. (1984). Total Internal Reflection Fluorescence. Annu. Rev. Biophys. Bioeng. 13, 247–268. 10.1146/annurev.bb.13.060184.001335 6378070

[B6] Axelrod D. (2008). Chapter 7 Total Internal Reflection Fluorescence Microscopy. Method Cel Biol. 89, 169–221. 10.1016/s0091-679x(08)00607-9 19118676

[B7] Balch W. E. Dunphy W. G. Braell W. A. Rothman J. E. (1984). Reconstitution of the Transport of Protein between Successive Compartments of the Golgi Measured by the Coupled Incorporation of N-Acetylglucosamine. Cell 39, 405–416. 10.1016/0092-8674(84)90019-9 6498939

[B8] Bao H. Das D. Courtney N. A. Jiang Y. Briguglio J. S. Lou X. (2018). Dynamics and Number of Trans-SNARE Complexes Determine Nascent Fusion Pore Properties. Nature 554, 260–263. 10.1038/nature25481 29420480 PMC5808578

[B9] Barg S. Olofsson C. S. Schriever-Abeln J. Wendt A. Gebre-Medhin S. Renström E. (2002). Delay between Fusion Pore Opening and Peptide Release from Large Dense-Core Vesicles in Neuroendocrine Cells. Neuron 33, 287–299. 10.1016/s0896-6273(02)00563-9 11804575

[B10] Bello O. D. Auclair S. M. Rothman J. E. Krishnakumar S. S. (2016). Using ApoE Nanolipoprotein Particles to Analyze SNARE-Induced Fusion Pores. Langmuir 32, 3015–3023. 10.1021/acs.langmuir.6b00245 26972604 PMC4946868

[B11] Bowen M. E. Weninger K. Brunger A. T. Chu S. (2004). Single Molecule Observation of Liposome-Bilayer Fusion Thermally Induced by Soluble N-Ethyl Maleimide Sensitive-Factor Attachment Protein Receptors (SNAREs). Biophysical J. 87, 3569–3584. 10.1529/biophysj.104.048637 PMC1304822 15347585

[B12] Breckenridge L. J. Almers W. (1987). Currents through the Fusion Pore that Forms during Exocytosis of a Secretory Vesicle. Nature 328, 814–817. 10.1038/328814a0 2442614

[B13] Bulow U. Govindan R. Munro J. B. (2020). Acidic pH Triggers Lipid Mixing Mediated by Lassa Virus GP. Viruses 12. 10.3390/v12070716 PMC7411951 32630688

[B14] Chang C.-W. Chiang C.-W. Jackson M. B. (2017). Fusion Pores and Their Control of Neurotransmitter and Hormone Release. J. Gen. Physiol. 149, 301–322. 10.1085/jgp.201611724 28167663 PMC5339513

[B15] Chanturiya A. Chernomordik L. V. Zimmerberg J. (1997). Flickering Fusion Pores Comparable with Initial Exocytotic Pores Occur in Protein-free Phospholipid Bilayers. Proc. Natl. Acad. Sci. 94, 14423–14428. 10.1073/pnas.94.26.14423 9405628 PMC25008

[B16] Chapochnikov N. M. Takago H. Huang C.-H. Pangršič T. Khimich D. Neef J. (2014). Uniquantal Release through a Dynamic Fusion Pore Is a Candidate Mechanism of Hair Cell Exocytosis. Neuron. 83, 1389–1403. 10.1016/j.neuron.2014.08.003 25199706

[B17] Chernomordik L. V. Kozlov M. M. (2008). Mechanics of Membrane Fusion. Nat. Struct. Mol. Biol. 15, 675–683. 10.1038/nsmb.1455 18596814 PMC2548310

[B18] Chiang H.-C. Shin W. Zhao W.-D. Hamid E. Sheng J. Baydyuk M. (2014). Post-fusion Structural Changes and Their Roles in Exocytosis and Endocytosis of Dense-Core Vesicles. Nat. Commun. 5, 3356. 10.1038/ncomms4356 24561832 PMC4267856

[B19] Cohen F. S. Melikyan G. B. (2004). The Energetics of Membrane Fusion from Binding, through Hemifusion, Pore Formation, and Pore Enlargement. J. Membr. Biol. 199, 1–14. 10.1007/s00232-004-0669-8 15366419

[B20] Costello D. A. Hsia C.-Y. Millet J. K. Porri T. Daniel S. (2013). Membrane Fusion-Competent Virus-like Proteoliposomes and Proteinaceous Supported Bilayers Made Directly from Cell Plasma Membranes. Langmuir 29, 6409–6419. 10.1021/la400861u 23631561

[B21] Crane J. M. Kiessling V. Tamm L. K. (2005). Measuring Lipid Asymmetry in Planar Supported Bilayers by Fluorescence Interference Contrast Microscopy. Langmuir 21, 1377–1388. 10.1021/la047654w 15697284

[B22] Das D. Bao H. Courtney K. C. Wu L. Chapman E. R. (2020). Resolving Kinetic Intermediates during the Regulated Assembly and Disassembly of Fusion Pores. Nat. Commun. 11, 231. 10.1038/s41467-019-14072-7 31932584 PMC6957489

[B23] de Toledo G. A. Fernández-Chacón R. Fernández J. M. (1993). Release of Secretory Products during Transient Vesicle Fusion. Nature 363, 554–558. 10.1038/363554a0 8505984

[B24] Diao J. Su Z. Ishitsuka Y. Lu B. Lee K. S. Lai Y. (2010). A Single-Vesicle Content Mixing Assay for SNARE-Mediated Membrane Fusion. Nat. Commun. 1, 54. 10.1038/ncomms1054 20975723 PMC3518844

[B25] Diao J. Grob P. Cipriano D. J. Kyoung M. Zhang Y. Shah S. (2013). Synaptic Proteins Promote Calcium-Triggered Fast Transition from point Contact to Full Fusion. elife 1, e00109. 10.7554/eLife.00109 PMC3514886 23240085

[B26] Domanska M. K. Kiessling V. Stein A. Fasshauer D. Tamm L. K. (2010). Single Vesicle Millisecond Fusion Kinetics Reveals Number of SNARE Complexes Optimal for Fast SNARE-Mediated Membrane Fusion. J. Biol. Chem. 285, 11753. 10.1074/jbc.a109.047381 PMC2797286 19759010

[B27] Dudzinski N. R. Wu Z. Karatekin E. (2018). “A Nanodisk-Cell Fusion Assay with Single-Pore Sensitivity and Sub-millisecond Time Resolution,” in SNAREs: Methods and Protocols. Editor Fratti R. (Springer Science+Business Media, LLC, part of Springer Nature). 10.1007/978-1-4939-8760-3_17 PMC6335040 30317511

[B28] Dudzinski N. R. Wu Z. Karatekin E. (2019). A Nanodisc-Cell Fusion Assay with Single-Pore Sensitivity and Sub-millisecond Time Resolution. Methods Mol. Biol. 1860, 263–275. 10.1007/978-1-4939-8760-3_17 30317511 PMC6335040

[B29] Edelstein A. D. Tsuchida M. A. Amodaj N. Pinkard H. Vale R. D. Stuurman N. (2014). Advanced Methods of Microscope Control Using μManager Software. J. Biol. Methods 1. 10.14440/jbm.2014.36 PMC4297649 25606571

[B30] Fix M. Melia T. J. Jaiswal J. K. Rappoport J. Z. You D. Sollner T. H. (2004). Imaging Single Membrane Fusion Events Mediated by SNARE Proteins. Proc. Natl. Acad. Sci. 101, 7311–7316. 10.1073/pnas.0401779101 15123811 PMC409915

[B31] Floyd D. L. Ragains J. R. Skehel J. J. Harrison S. C. van Oijen A. M. (2008). Single-particle Kinetics of Influenza Virus Membrane Fusion. Pnas 105, 15382–15387. 10.1073/pnas.0807771105 18829437 PMC2556630

[B32] Gandhi S. P. Stevens C. F. (2003). Three Modes of Synaptic Vesicular Recycling Revealed by Single-Vesicle Imaging. Nature 423, 607–613. 10.1038/nature01677 12789331

[B33] Gao L. Lipowsky R. Shillcock J. (2008). Tension-induced Vesicle Fusion: Pathways and Pore Dynamics. Soft Matter 4, 1208–1214. 10.1039/b801407h 32907263

[B34] Harrison S. C. (2008). Viral Membrane Fusion. Nat. Struct. Mol. Biol. 15, 690–698. 10.1038/nsmb.1456 18596815 PMC2517140

[B35] Hastoy B. Clark A. Rorsman P. Lang J. (2017). Fusion Pore in Exocytosis: More Than an Exit Gate? A β-cell Perspective. Cell Calcium 68, 45–61. 10.1016/j.ceca.2017.10.005 29129207

[B36] He L. Wu X.-S. Mohan R. Wu L.-G. (2006). Two Modes of Fusion Pore Opening Revealed by Cell-Attached Recordings at a Synapse. Nature 444, 102–105. 10.1038/nature05250 17065984

[B37] Hernandez J. M. Stein A. Behrmann E. Riedel D. Cypionka A. Farsi Z. (2012). Membrane Fusion Intermediates via Directional and Full Assembly of the SNARE Complex. Science 336, 1581–1584. 10.1126/science.1221976 22653732 PMC3677693

[B38] Hung W.-C. Lee M.-T. Chen F.-Y. Huang H. W. (2007). The Condensing Effect of Cholesterol in Lipid Bilayers. Biophysical J. 92, 3960–3967. 10.1529/biophysj.106.099234 PMC1868968 17369407

[B39] Ivanovic T. Choi J. L. Whelan S. P. van Oijen A. M. Harrison S. C. (2013). Influenza-virus Membrane Fusion by Cooperative Fold-Back of Stochastically Induced Hemagglutinin Intermediates. Elife 2, e00333. 10.7554/eLife.00333 23550179 PMC3578201

[B40] Jackson M. B. Chapman E. R. (2008). The Fusion Pores of Ca2+-Triggered Exocytosis. Nat. Struct. Mol. Biol. 15, 684–689. 10.1038/nsmb.1449 18596819 PMC2914174

[B41] Karatekin E. Rothman J. E. (2012). Fusion of Single Proteoliposomes with Planar, Cushioned Bilayers in Microfluidic Flow Cells. Nat. Protoc. 7, 903–920. 10.1038/nprot.2012.019 22517259 PMC3747733

[B42] Karatekin E. Di Giovanni J. Iborra C. Coleman J. O'Shaughnessy B. Seagar M. (2010). A Fast, Single-Vesicle Fusion Assay Mimics Physiological SNARE Requirements. Proc. Natl. Acad. Sci. USA. 107, 3517–3521. 10.1073/pnas.0914723107 20133592 PMC2840481

[B43] Karatekin E. (2018). Toward a Unified Picture of the Exocytotic Fusion Pore. FEBS Lett. 592, 3563–3585. 10.1002/1873-3468.13270 30317539 PMC6353554

[B44] Kiessling V. Domanska M. K. Tamm L. K. (2010). Single SNARE-Mediated Vesicle Fusion Observed *In Vitro* by Polarized TIRFM. Biophysical J. 99, 4047–4055. 10.1016/j.bpj.2010.10.022 PMC3000493 21156148

[B45] Kiessling V. Liang B. Kreutzberger A. J. Tamm L. K. (2017). Planar Supported Membranes with Mobile SNARE Proteins and Quantitative Fluorescence Microscopy Assays to Study Synaptic Vesicle Fusion. Front. Mol. Neurosci. 10, 72. 10.3389/fnmol.2017.00072 28360838 PMC5352703

[B46] Klyachko V. A. Jackson M. B. (2002). Capacitance Steps and Fusion Pores of Small and Large-Dense-Core Vesicles in Nerve Terminals. Nature 418, 89–92. 10.1038/nature00852 12097912

[B47] Kreutzberger A. J. B. Kiessling V. Liang B. Seelheim P. Jakhanwal S. Jahn R. (2017). Reconstitution of Calcium-Mediated Exocytosis of Dense-Core Vesicles. Sci. Adv. 3, e1603208. 10.1126/sciadv.1603208 28776026 PMC5517108

[B48] Kreutzberger A. J. B. Kiessling V. Stroupe C. Liang B. Preobraschenski J. Ganzella M. (2019). *In Vitro* fusion of Single Synaptic and Dense Core Vesicles Reproduces Key Physiological Properties. Nat. Commun. 10, 3904. 10.1038/s41467-019-11873-8 31467284 PMC6715626

[B49] Kyoung M. Zhang Y. Diao J. Chu S. Brunger A. T. (2013). Studying Calcium-Triggered Vesicle Fusion in a Single Vesicle-Vesicle Content and Lipid-Mixing System. Nat. Protoc. 8, 1–16. 10.1038/nprot.2012.134 23222454 PMC3566647

[B50] Lai Y. Diao J. Liu Y. Ishitsuka Y. Su Z. Schulten K. (2013). Fusion Pore Formation and Expansion Induced by Ca2+and Synaptotagmin 1. Proc. Natl. Acad. Sci. USA. 110, 1333–1338. 10.1073/pnas.1218818110 23300284 PMC3557091

[B51] Lai Y. Diao J. Cipriano D. J. Zhang Y. Pfuetzner R. A. Padolina M. S. (2014). Complexin Inhibits Spontaneous Release and Synchronizes Ca2+-Triggered Synaptic Vesicle Fusion by Distinct Mechanisms. Elife 3, e03756. 10.7554/eLife.03756 25122624 PMC4130161

[B52] Lai Y. Choi U. B. Leitz J. Rhee H. J. Lee C. Altas B. (2017). Molecular Mechanisms of Synaptic Vesicle Priming by Munc13 and Munc18. Neuron 95, 591–607. 10.1016/j.neuron.2017.07.004 28772123 PMC5747255

[B53] Lindau M. Alvarez de Toledo G. (2003). The Fusion Pore. Biochim. Biophys. Acta (Bba) - Mol. Cel Res. 1641, 167–173. 10.1016/s0167-4889(03)00085-5 12914957

[B54] Lisman J. E. Raghavachari S. Tsien R. W. (2007). The Sequence of Events that Underlie Quantal Transmission at central Glutamatergic Synapses. Nat. Rev. Neurosci. 8, 597–609. 10.1038/nrn2191 17637801

[B55] Liu T. Tucker W. C. Bhalla A. Chapman E. R. Weisshaar J. C. (2005). SNARE-driven, 25-millisecond Vesicle Fusion *In Vitro* . Biophysical J. 89, 2458–2472. 10.1529/biophysj.105.062539 PMC1366745 16055544

[B56] Malinin V. S. Frederik P. Lentz B. R. (2002). Osmotic and Curvature Stress Affect PEG-Induced Fusion of Lipid Vesicles but Not Mixing of Their Lipids. Biophysical J. 82, 2090–2100. 10.1016/s0006-3495(02)75556-2 PMC1302003 11916865

[B57] Martens S. McMahon H. T. (2008). Mechanisms of Membrane Fusion: Disparate Players and Common Principles. Nat. Rev. Mol. Cel Biol. 9, 543–556. 10.1038/nrm2417 18496517

[B58] Melikyan G. B. Niles W. D. Peeples M. E. Cohen F. S. (1993). Influenza Hemagglutinin-Mediated Fusion Pores Connecting Cells to Planar Membranes: Flickering to Final Expansion. J. Gen. Physiol. 102, 1131–1149. 10.1085/jgp.102.6.1131 8133242 PMC2229191

[B59] Melikyan G. B. Niles W. D. Cohen F. S. (1993). Influenza Virus Hemagglutinin-Induced Cell-Planar Bilayer Fusion: Quantitative Dissection of Fusion Pore Kinetics into Stages. J. Gen. Physiol. 102, 1151–1170. 10.1085/jgp.102.6.1151 8133243 PMC2229187

[B60] Melikyan G. B. Niles W. D. Ratinov V. A. Karhanek M. Zimmerberg J. Cohen F. S. (1995). Comparison of Transient and Successful Fusion Pores Connecting Influenza Hemagglutinin Expressing Cells to Planar Membranes. J. Gen. Physiol. 106, 803–819. 10.1085/jgp.106.5.803 8648293 PMC2229290

[B61] Monck J. R. Fernandez J. M. (1994). The Exocytotic Fusion Pore and Neurotransmitter Release. Neuron 12, 707–716. 10.1016/0896-6273(94)90325-5 7909233

[B62] Nikolaus J. Karatekin E. (2016). SNARE-mediated Fusion of Single Proteoliposomes with Tethered Supported Bilayers in a Microfluidic Flow Cell Monitored by Polarized TIRF Microscopy. J. Vis. Exp. 114. 10.3791/54349 PMC5091947 27585113

[B63] Pawlu C. DiAntonio A. Heckmann M. (2004). Postfusional Control of Quantal Current Shape. Neuron 42, 607–618. 10.1016/s0896-6273(04)00269-7 15157422

[B64] Rorsman P. Ashcroft F. M. (2018). Pancreatic β-Cell Electrical Activity and Insulin Secretion: Of Mice and Men. Physiol. Rev. 98, 117–214. 10.1152/physrev.00008.2017 29212789 PMC5866358

[B65] Rothman J. E. (2014). The Principle of Membrane Fusion in the Cell (Nobel Lecture). Angew. Chem. Int. Ed. 53, 12676–12694. 10.1002/anie.201402380 25087728

[B66] Schneider C. A. Rasband W. S. Eliceiri K. W. (2012). NIH Image to ImageJ: 25 Years of Image Analysis. Nat. Methods 9, 671–675. 10.1038/nmeth.2089 22930834 PMC5554542

[B67] Sharma S. Lindau M. (2018). The Fusion Pore, 60 Years after the First Cartoon. FEBS Lett. 592, 3542–3562. 10.1002/1873-3468.13160 29904915 PMC6231997

[B68] Shi L. Shen Q.-T. Kiel A. Wang J. Wang H.-W. Melia T. J. (2012). SNARE Proteins: One to Fuse and Three to Keep the Nascent Fusion Pore Open. Science 335, 1355–1359. 10.1126/science.1214984 22422984 PMC3736847

[B69] Shin W. Ge L. Arpino G. Villarreal S. A. Hamid E. Liu H. (2018). Visualization of Membrane Pore in Live Cells Reveals a Dynamic-Pore Theory Governing Fusion and Endocytosis. Cell 173, 934–945. 10.1016/j.cell.2018.02.062 29606354 PMC5935532

[B70] Smith E. A. Weisshaar J. C. (2011). Docking, Not Fusion, as the Rate-Limiting Step in a SNARE-Driven Vesicle Fusion Assay. Biophysical J. 100, 2141–2150. 10.1016/j.bpj.2011.03.015 PMC3149229 21539781

[B71] Smith M. B. Karatekin E. Gohlke A. Mizuno H. Watanabe N. Vavylonis D. (2011). Interactive, Computer-Assisted Tracking of Speckle Trajectories in Fluorescence Microscopy: Application to Actin Polymerization and Membrane Fusion. Biophysical J. 101, 1794–1804. 10.1016/j.bpj.2011.09.007 PMC3183794 21961607

[B72] Spruce A. E. Iwata A. White J. M. Almers W. (1989). Patch Clamp Studies of Single Cell-Fusion Events Mediated by a Viral Fusion Protein. Nature 342, 555–558. 10.1038/342555a0 2586627

[B73] Staal R. G. W. Mosharov E. V. Sulzer D. (2004). Dopamine Neurons Release Transmitter via a Flickering Fusion Pore. Nat. Neurosci. 7, 341–346. 10.1038/nn1205 14990933

[B74] Stratton B. S. Warner J. M. Wu Z. Nikolaus J. Wei G. Wagnon E. (2016). Cholesterol Increases the Openness of SNARE-Mediated Flickering Fusion Pores. Biophysical J. 110, 1538–1550. 10.1016/j.bpj.2016.02.019 PMC4833774 27074679

[B75] Taraska J. W. Perrais D. Ohara-Imaizumi M. Nagamatsu S. Almers W. (2003). Secretory Granules Are Recaptured Largely Intact after Stimulated Exocytosis in Cultured Endocrine Cells. Proc. Natl. Acad. Sci. 100, 2070–2075. 10.1073/pnas.0337526100 12538853 PMC149960

[B76] Tran V. S. Huet S. Fanget I. Cribier S. Henry J.-P. Karatekin E. (2007). Characterization of Sequential Exocytosis in a Human Neuroendocrine Cell Line Using Evanescent Wave Microscopy and "virtual Trajectory" Analysis. Eur. Biophys. J. 37, 55–69. 10.1007/s00249-007-0161-3 17440716

[B77] van den Bogaart G. Holt M. G. Bunt G. Riedel D. Wouters F. S. Jahn R. (2010). One SNARE Complex Is Sufficient for Membrane Fusion. Nat. Struct. Mol. Biol. 17, 358–364. 10.1038/nsmb.1748 20139985 PMC2924150

[B78] Verstreken P. Kjaerulff O. Lloyd T. E. Atkinson R. Zhou Y. Meinertzhagen I. A. (2002). Endophilin Mutations Block Clathrin-Mediated Endocytosis but Not Neurotransmitter Release. Cell 109, 101–112. 10.1016/s0092-8674(02)00688-8 11955450

[B79] Weber T. Zemelman B. V. McNew J. A. Westermann B. Gmachl M. Parlati F. (1998). SNAREpins: Minimal Machinery for Membrane Fusion. Cell 92, 759–772. 10.1016/s0092-8674(00)81404-x 9529252

[B80] Wu Z. Auclair S. M. Bello O. Vennekate W. Dudzinski N. R. Krishnakumar S. S. (2016). Nanodisc-cell Fusion: Control of Fusion Pore Nucleation and Lifetimes by SNARE Protein Transmembrane Domains. Sci. Rep. 6, 27287. 10.1038/srep27287 27264104 PMC4893671

[B81] Wu Z. Bello O. D. Thiyagarajan S. Auclair S. M. Vennekate W. Krishnakumar S. S. (2017). Dilation of Fusion Pores by Crowding of SNARE Proteins. Elife 6, e22964. 10.7554/eLife.22964 28346138 PMC5404929

[B82] Wu Z. Dharan N. McDargh Z. A. Thiyagarajan S. O'Shaughnessy B. Karatekin E. (2021). The Neuronal Calcium Sensor Synaptotagmin-1 and SNARE Proteins Cooperate to Dilate Fusion Pores. Elife 10. 10.7554/eLife.68215 PMC8294851 34190041

[B83] Xu W. Wang J. Rothman J. E. Pincet F. (2015). Accelerating SNARE‐Mediated Membrane Fusion by DNA-Lipid Tethers. Angew. Chem. Int. Ed. 54, 14388–14392. 10.1002/anie.201506844 PMC4888371 26439984

[B84] Yang Y. Wu Z. Wang L. Zhou K. Xia K. Xiong Q. (2021). Sorting Sub-150-nm Liposomes of Distinct Sizes by DNA-brick-assisted Centrifugation. Nat. Chem. 13, 335–342. 10.1038/s41557-021-00667-5 33785892 PMC8049973

[B85] Zhang Z. Jackson M. B. (2010). Membrane Bending Energy and Fusion Pore Kinetics in Ca2+-Triggered Exocytosis. Biophysical J. 98, 2524–2534. 10.1016/j.bpj.2010.02.043 PMC2877347 20513396

[B86] Zhao Y. Fang Q. Herbst A. D. Berberian K. N. Almers W. Lindau M. (2013). Rapid Structural Change in Synaptosomal-Associated Protein 25 (SNAP25) Precedes the Fusion of Single Vesicles with the Plasma Membrane in Live Chromaffin Cells. Proc. Natl. Acad. Sci. 110, 14249–14254. 10.1073/pnas.1306699110 23940346 PMC3761627

[B87] Zhou Z. Misler S. Chow R. H. (1996). Rapid Fluctuations in Transmitter Release from Single Vesicles in Bovine Adrenal Chromaffin Cells. Biophysical J. 70, 1543–1552. 10.1016/s0006-3495(96)79718-7 PMC1225082 8785312

