# Supplementary material for: Optimal Detection of Fusion Pore Dynamics Using Polarized Total Internal Reflection Fluorescence Microscopy
Source: Front Mol Biosci. 2021 Nov 10;8:740408. doi: 10.3389/fmolb.2021.740408 (PMC8631473; doi:10.3389/fmolb.2021.740408)
Supplement: Supplementary file 1 [file Presentation1.pdf]

SUPPLEMENTARY INFORMATION

FOR

OPTIMAL DETECTION OF FUSION PORE DYNAMICS USING POLARIZED TOTAL INTERNAL REFLECTION FLUORESCENCE MICROSCOPY

Joerg Nikolaus[1,2,†,§], Kasey Hancock[1,2,3,†,#], Maria Tsemperouli[1,2], David Baddeley[2,4,&,*], and Erdem Karatekin[1,2,5,6,*]

1 Cellular and Molecular Physiology, Yale University, New Haven, CT

2 Nanobiology Institute, Yale University, West Haven, CT

3 Integrated Physical and Engineering Biology Program, Yale University, New Haven, CT

4 Cell Biology, Yale University

5 Molecular Biophysics and Biochemistry, Yale University, New Haven, CT

6 Saints-Pères Paris Institute for the Neurosciences (SPPIN), Université de Paris, Centre National de la Recherche Scientifique (CNRS) UMR 8003, Paris, France

[§] Present address: Director, Yale University West Campus Imaging Core

[#] Present address: Electrical Engineering, Yale University

[&] Present address: Auckland Bioengineering Institute, University of Auckland, New Zealand

[†] These authors contributed equally.

* correspondence should be addressed to DB: d.baddeley@auckland.ac.nz or EK: erdem.karatekin@yale.edu

SUPPLEMENTARY FIGURE CAPTIONS

Figure S1. **Schematic of the polarized TIRF microscope.** Polarized outputs from lasers are fiber coupled, then combined into a single fiber using a wavelength division multiplexer (**WDM**) that preserves polarization (OZ Optics, Ottawa, Canada). The output of the 561 nm laser line is controlled by an acousto-optical modulator (AOTF). A manual rotation mount (**RM**) is used to rotate the excitation field polarization. The beam leaving the fiber is expanded (**L1, L2**) before passing through a diaphragm (**D**) and reflecting off mirror **M1**. A motorized actuator (**MA**) rotates the mirror to adjust the position of the beam at the back focal plane (BFP) of the objective (Obj.) and set the evanescent field depth reproducibly. Before reaching the BFP, the beam passes through a tube lens (**L3**) and a filter cube with a narrow-band excitation clean-up filter (**Ex**) and a dichroic mirror (**DM**). After exciting the sample, light is collected through the same objective and passes through an emission filter (**Em**) before being projected onto an EM-CCD chip using mirrors **M2** and **M3**. The sample is a SBL formed on a glass coverslip interacting with fluorescently-labeled SUVs introduced through a microfluidic system. A buffer containing SUVs is aspirated into a microfluidic channel using a syringe pump (not shown for clarity). The channels are made by adhering a polydimethylsiloxane (PDMS) block onto the coverslip. Temperature is controlled using a heated microscope stage insert. The setup is controlled using the open-source program micro-manager[63].

Figure S2. **Distribution of single lipid fluorescence intensities and variation of I_{lip} as a function of excitation polarization. A.** Example of trajectories of single fluorescent lipids tracked after a fusion event using Speckle TrackerJ[84]. Image size is 16 µm × 16 µm. The sample was excited using s-pol light. The vesicle membrane was labeled with 1% LR-PE which transferred to the SBL and diffused away from the fusion site upon fusion. **B.** Distribution of single fluorescent lipid intensities (s-pol). For each fluorescent lipid that was tracked as in A, the mean pixel value in a 3 pixel by 3 pixel area around the spot's tracked center of mass was calculated. This value was corrected by subtracting the local mean intensity, defined as the mean intensity in a 30 pixels by 30 pixels box ($8\ \mu m\ \times 8\ \mu m$) centered at the docking site and averaged for multiple frames prior to docking. It was checked that no other SUVs docked in this region of interest. The distribution of corrected single lipid intensities was fitted to a Gaussian distribution to estimate the average single lipid intensity $I_{lip} = 117\ a.u.$ with std. deviation 30.8 a.u. (23 individual lipids were tracked for 4-19 frames. All points were pooled (n=185) and intensity >200 a.u. were excluded). **C.** SBL fluorescence TIRF images at the indicated excitation polarization angles. An SBL was formed by deposition of vesicles doped with 0.5% LR-PE. Four different polarizations are shown. Images are 136 µm × 136 µm. **D.** Percent change in mean fluorescence intensity, F, as a function of excitation polarization angle θ, i.e. $100(F(\theta) - F_0)/F_0$, where F_0 is the intensity at $\theta = 0°$. A total of 7 areas in 2 independent SBLs were measured as in C, at the indicated θ. The continuous line is a sinusoidal fit to the data $f(\theta) = a\ \sin(\omega\,\theta + \phi) + c$, with best fit parameters (and 95% confidence intervals): $a = 9.23\ (8.77, 9.69)$, $c = -7.19\ (-7.67, -6.71)$, $\omega = 0.044\ (0.041, 0.048)$, $\phi = 0.93\ (1.1, 0.78)$ ($R^2 = 0.998$). In C,D, uniformly-labeled SBLs at different polarization angles are imaged;

excitation dipoles of LR-PE molecules are confined to the SBL and the excitation polarization angle is varied. Note that these measurements are *not* equivalent to measurements of intensity changes that occur when the fluorophores are transferred from a SUV into the SBL upon fusion, where the excitation polarization is fixed while the distribution of excitation dipole orientations changes.

Figure S3. **Overview of fit model.** Our model is based on the assumption that, upon fusion lipids transfer at a constant rate from the vesicle into the bilayer (A) giving rise to an exponential decay in vesicle lipid content (modelled as $\tau_{release}$) and a concomitant increase in bilayer concentration. From this we calculate the *total* fluorescence from each component (B), including the enhancement (or reduction), $G = 1/\lambda_{TIRF}$, due to polarization, evanescent field, and dequenching which occurs as lipids transfer to the bilayer, and a bleaching component. As we expect the vesicle (blue) and bilayer (orange) components of the signal to have different spatial and temporal characteristics (C) we evaluate the amount of the total fluorescence we expect to be contained in several radial ROIs (D). This leads to a family of model curves (E – simulated for $\tau_{release}$ = 2 frames, τ_{bleach}=5 frames, $D = 0.1$ pixel^2/frame and $\lambda_{TIRF} = 0.5$) from which diffusion and release behaviors can be independently extracted (see Materials and Methods section for details). Panels F and G show example fits of the model to real experimental data for the s-pol ($\theta = 0°$) and p-pol ($\theta = 90°$) cases (frame interval = 18.3 ms).

Figure S4. **Data acquisition and analysis pipeline.** Raw movies are first analyzed using the SpeckleTrackerJ plugin for ImageJ to visually identify and label individual fusion events. A given vesicle is tracked from the first frame in which it docks until the frame just preceding fusion, providing τ_{dock}. Event locations are saved in a text file (.csv) which are fed together with the original movies to python-microscopy (PYME) to extract regions of interests (ROIs) around docking and fusion events for fitting the traces to extract the parameters λ_{TIRF}, τ_{bleach}, D_{lip}, and $\tau_{release}$. The vesicle intensity just after docking, I_{dock} was estimated directly from the intensity change upon docking in the largest radial profile. Every fit is visually assessed for quality control. Fits passing the quality control are saved in a text (.csv) file. Single lipid intensity I_{lip} is estimated from the raw movies by localizing individual fluorescent lipids and integrating the pixel values in a 3 pixels × 3 pixels box around the spots using SpeckeTrackerJ/ImageJ. Using the known labeling density, vesicle area A_{ves} (and radius R_{ves}) is calculated using eq. 2 by combining I_{lip} from single lipid analysis and intensity trace fitting parameters. Finally, comparing the actual release time $\tau_{release}$ to the release time expected from diffusion-limited release, $\tau_{ves} = A_{ves}/D_{lip}$, allows pore openness to be calculated (eq. 1). Parameter values are stored for individual fusion events. Note that single lipid analysis (dashed lines) is only required for initial experiments: once the dependence of λ_{TIRF} on R_{ves} is determined, this empirical relationship can be used to estimate R_{ves}, even if illumination intensities or integration times are changed.

Figure S5. Estimation of the purely polarization contribution to the intensity change as a lipid-dye is transferred from the SUV to the SBL upon fusion, for various excitation polarizations. (Related to Fig. 3). A. The relationship between the fluorescence intensity reduction factor λ_{TIRF} and docked SUV intensity I_{dock} normalized by single lipid intensity in the

SBL, I_{lip}, for $\theta = 30°$. 111 events analyzed. **B.** Vesicle radius, R_{ves}, as a function of the normalized docked SUV intensity, I_{dock}/I_{lip}. 111 events analyzed. **C**. Fluorescence intensity reduction factor λ_{TIRF} as a function of vesicle radius R_{ves}. 111 events analyzed. **D-F**. The same, for $\theta = 60°$. 67 events analyzed. **G-I.** Same quantities, for $\theta = 90°$ (p-pol). 73 events analyzed. For A, D, G, the red curve is a fit to an exponential, $\lambda_{fit} = a_1\ exp\left(-b_1\, I_{dock}/I_{lip}\right)$, with best-fit parameters (and 95 % confidence intervals) $a_1 = 0.60$ (0.54, 0.66) and $b_1 = 3.7 \times 10^{-4}$ (-5.1×10^{-4}, 1.2×10^{-3}) (A, $\theta = 30°$), $a_1 = 1.31$ (1.13, 1.50) and $b_1 = 2.0 \times 10^{-3}$ (7.9×10^{-4}, 3.1×10^{-3}) (D, $\theta = 60°$), and $a_1 = 1.52$ (1.34, 1.70) and $b_1 = 0.59 \times 10^{-3}$ (-$6.8\times 10^{-7}, 1.2 \times 10^{-3}$) (G, $\theta = 90°$) . For B, E, H, the red curves are fitted power laws, $R_{ves,fit} = a_2\ (I_{dock}/I_{SBL})^{b_2}$, with best fit parameters $a_2 = 2.08$ (1.56, 2.60) and $b_2 = 0.56$ (0.51, 0.62) (B, $\theta = 30°$), $a_2 = 1.08$ (0.58, 1.57) and $b_2 = 0.64$ (0.55, 0.73) (G, $\theta = 60°$), and $a_2 = 1.10$ (0.69, 1.50) and $b_2 = 0.60$ (0.53, 0.66) (H, $\theta = 90°$). For C, F, I, the continuous red curves are exponential fits, $\lambda_{fit2} = a_1\ exp(-b_1\, R_{ves})$, with $a_1 = 0.81\ (0.70, 0.92)$; $b_1 = 0.014\ (0.020, 0.0079)$ (C, $\theta = 30°$), $a_1 = 1.93\ (1.66, 2.20)$; $b_1 = 0.029\ (0.022, 0.026)$ (F, $\theta = 60°$), and $a_1 = 2.18$ (1.86,2.50); $b_1 = 0.021\ (0.014, 0.028)$ (I, $\theta = 90°$). The red dashed lines are fits to the small R_{ves} values ($R_{ves} \leq 35$ nm) with the x-intercept constrained at the independently estimated evanescent field depth, $\delta_{TIRFM} = 78$ nm (see text and Materials and Methods). The y-intercepts are $\lambda^0_{TIRFM} = 0.83 \pm 0.05$, 1.5 ± 0.1, and $2.0 \pm .12$ for $\theta = 30°$, 60°, and 90°, respectively and represent our best estimates of the purely polarization contribution to the intensity change as a lipid-dye is transferred from a SUV into the SBL upon fusion. For a similar estimate at $\theta = 0°$ (s-pol), see Figure 3.

Figure S6. Effect of polarization on estimated parameters. A. Distribution of P_o values acquired using s-pol excitation, plotted as a probability density function (PDF). Bin size 0.05. 173 events analyzed. **B**. Distribution of docking-to-fusion delays at $\theta = 0°$. 173 events analyzed. **C-H**. Similar to A, B, for the polarization angles indicated. There were 129, 68, 63 events analyzed for the 30, 60, and 90 degree polarization plots, respectively. **I**. Distributions of P_o values for different polarizations plotted together as a cumulative density function (CDF), i.e. the probability that $P_o \geq$ than a given value. 433 events analyzed. **J.** Cumulative density functions of τ_{dock} values for different polarizations. 433 events analyzed. The values of P_o measured at different polarization angles were likely sampled from different distributions ($p = 0.004$, Kruskal-Wallis test). The distributions underlying the sampled values of τ_{dock} at different polarization angles were less likely to be different from one another ($p = 0.042$, Kruskal-Wallis test).

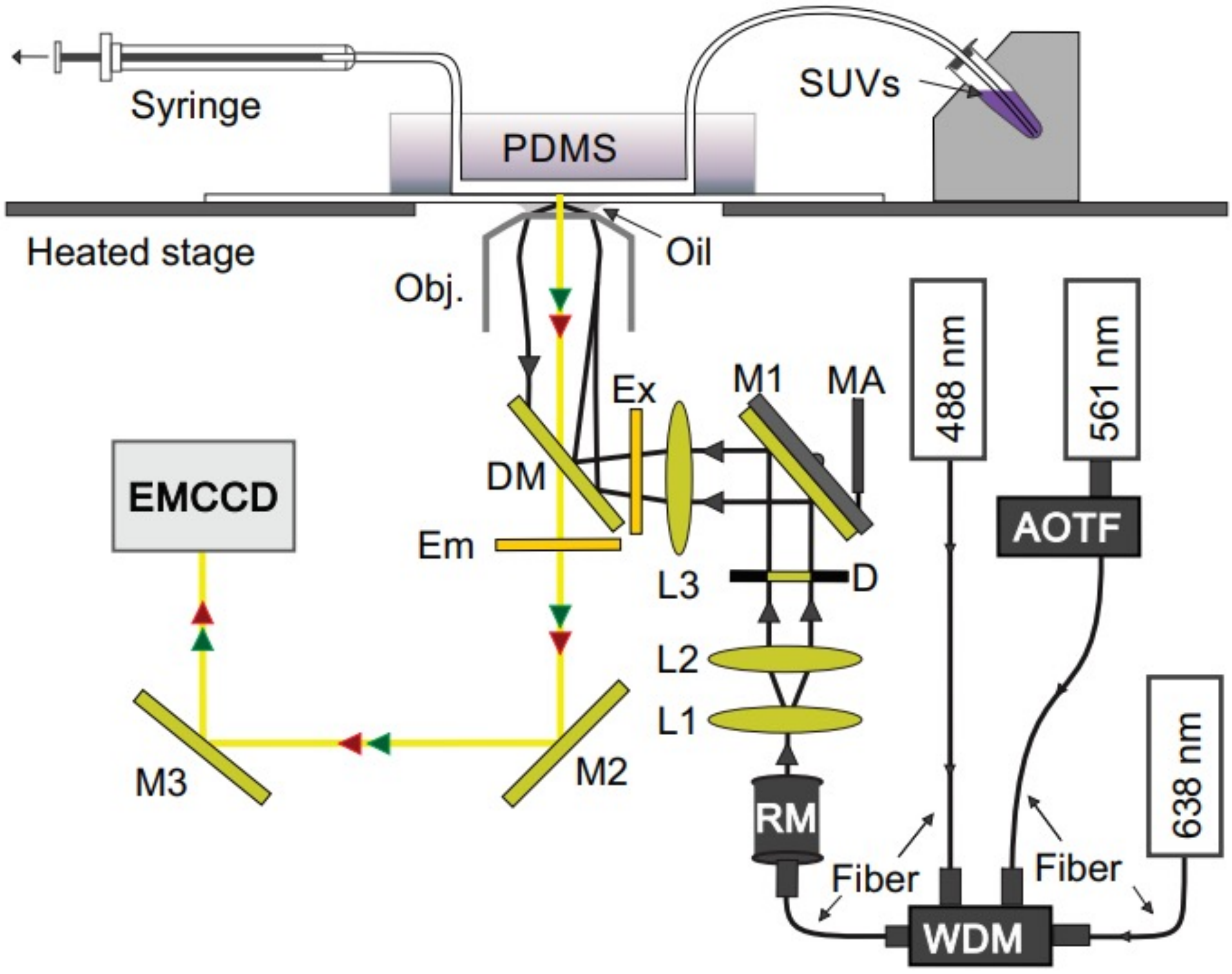
Syringe
PDMS
SUVs
Heated stage
Oil
Obj.
Ex
M1
MA
488 nm
561 nm
DM
EMCCD
AOTF
Em
L3
D
L2
L1
M3
M2
RM
Fiber
Fiber
638 nm
WDM

Figure S1

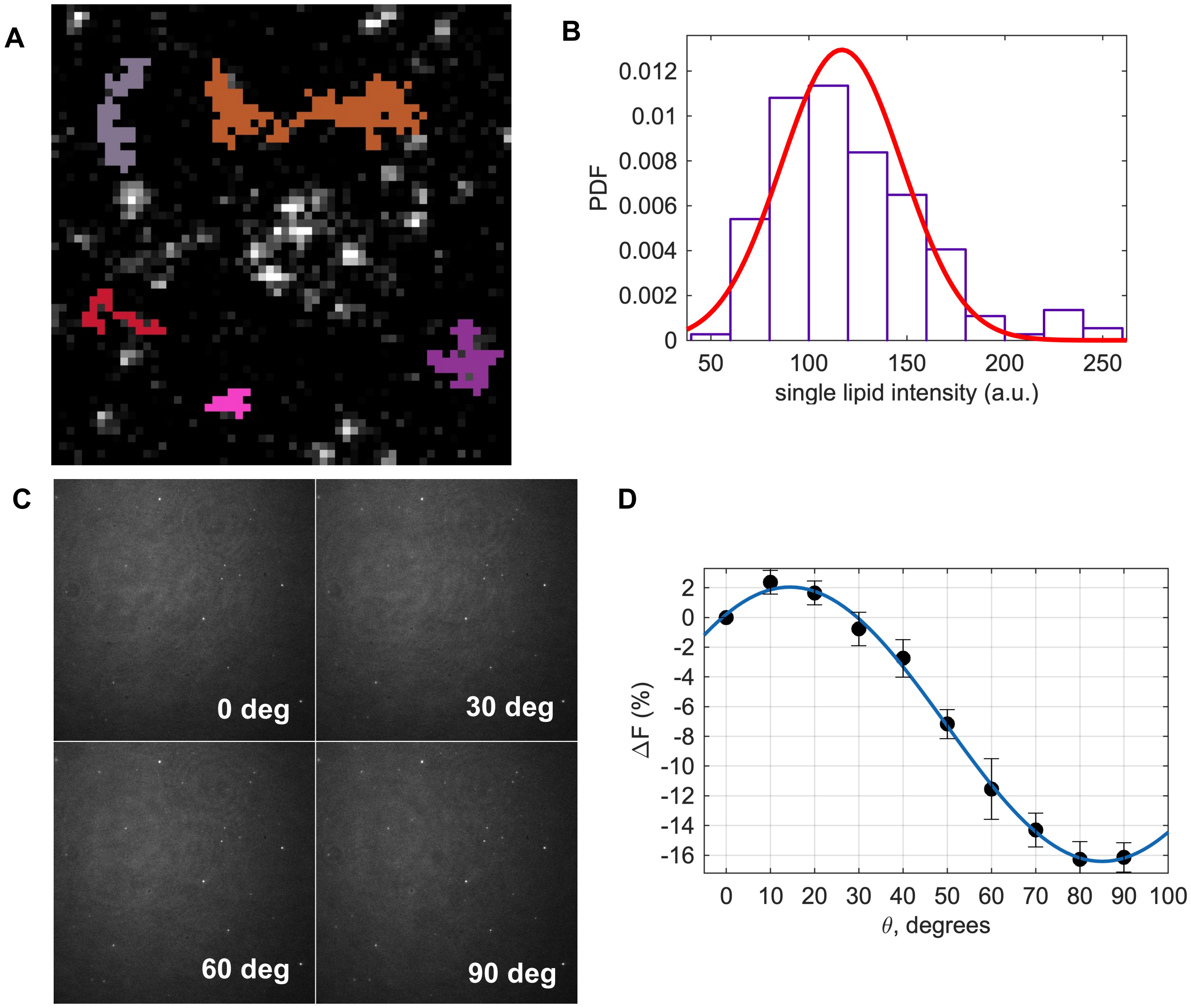
A
B
0.012
0.01
0.008
0.006
0.004
0.002
0
PDF
50
100
150
200
250
single lipid intensity (a.u.)
C
0 deg
30 deg
60 deg
90 deg
D
2
0
-2
-4
-6
-8
-10
-12
-14
-16
ΔF (%)
0
10
20
30
40
50
60
70
80
90
100
θ, degrees

Figure S2

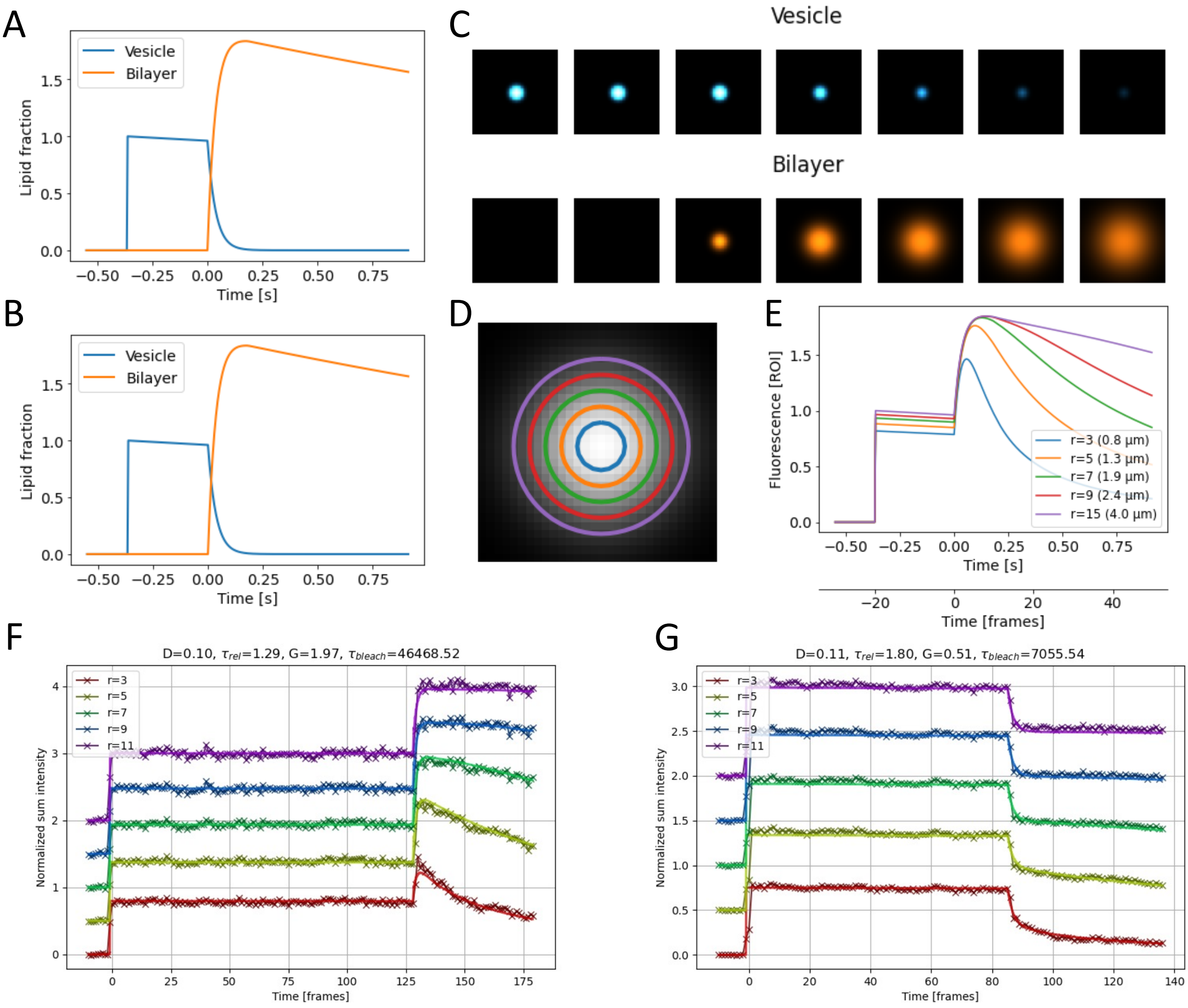
A
B
C
D
E
F
G
Vesicle
Bilayer
Lipid fraction
Time [s]
Fluorescence [ROI]
Time [frames]
r=3 (0.8 µm)
r=5 (1.3 µm)
r=7 (1.9 µm)
r=9 (2.4 µm)
r=15 (4.0 µm)
D=0.10, τ_{rel}=1.29, G=1.97, τ_{bleach}=46468.52
D=0.11, τ_{rel}=1.80, G=0.51, τ_{bleach}=7055.54
Normalized sum intensity
r=3
r=5
r=7
r=9
r=11

Figure S3

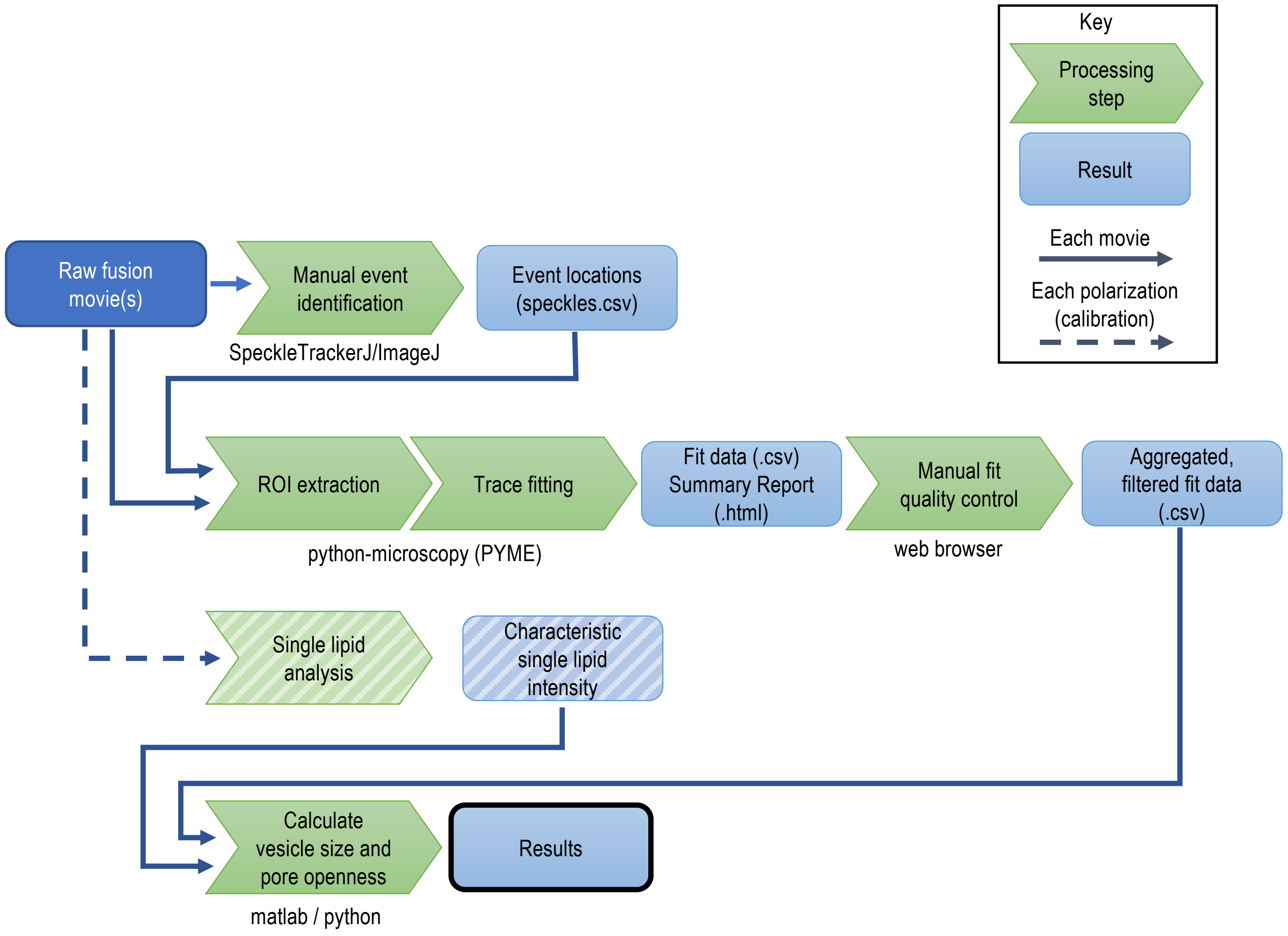
Key
Processing step
Result
Each movie
Each polarization (calibration)
Raw fusion movie(s)
Manual event identification
SpeckleTrackerJ/ImageJ
Event locations (speckles.csv)
ROI extraction
Trace fitting
python-microscopy (PYME)
Fit data (.csv) Summary Report (.html)
Manual fit quality control
web browser
Aggregated, filtered fit data (.csv)
Single lipid analysis
Characteristic single lipid intensity
Calculate vesicle size and pore openness
matlab / python
Results

Figure S4

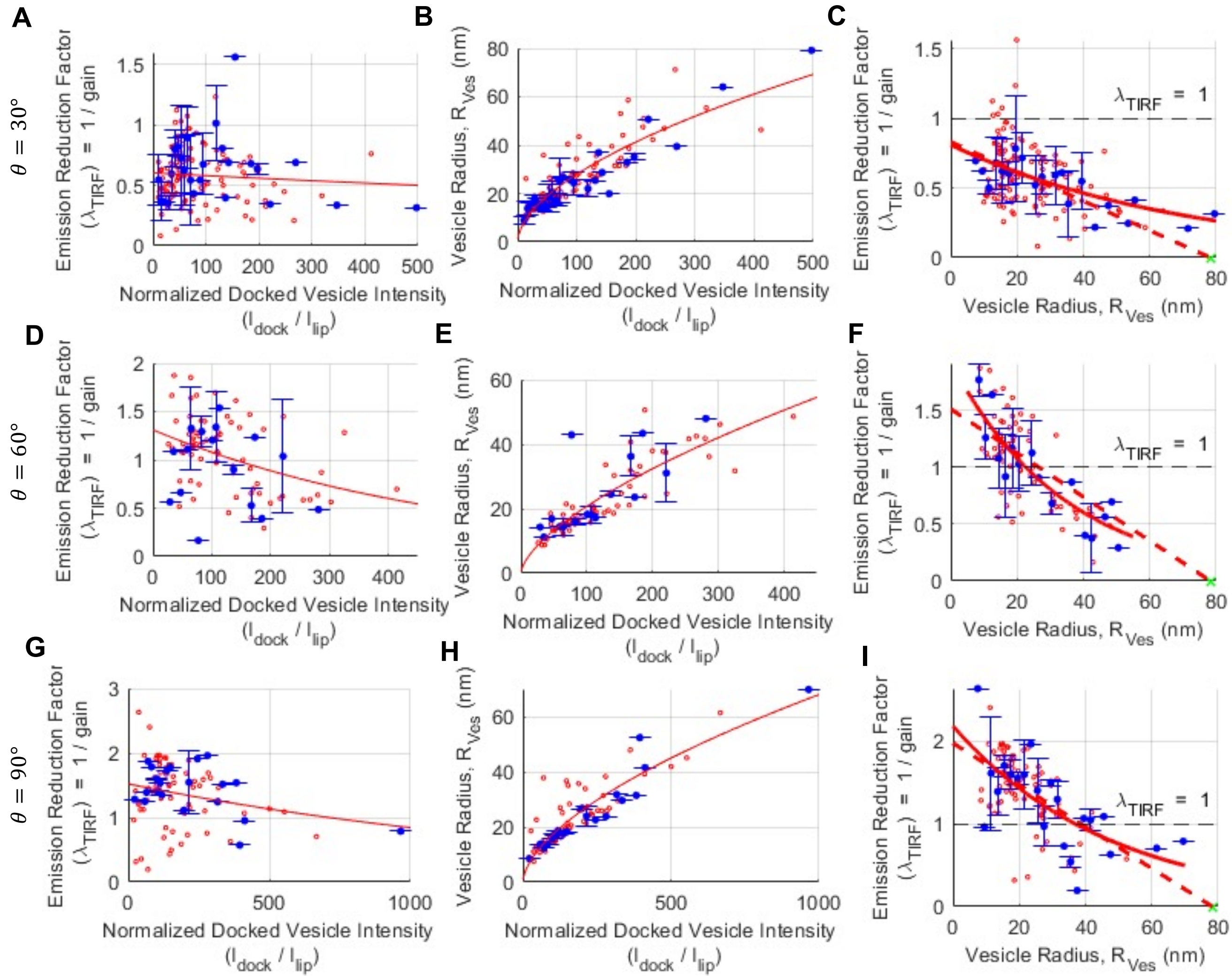
A
B
C
D
E
F
G
H
I
θ = 30°
θ = 60°
θ = 90°
Emission Reduction Factor
(λTIRF) = 1 / gain
Vesicle Radius, RVes (nm)
Normalized Docked Vesicle Intensity
(Idock / Ilip)
λTIRF = 1

Figure S5

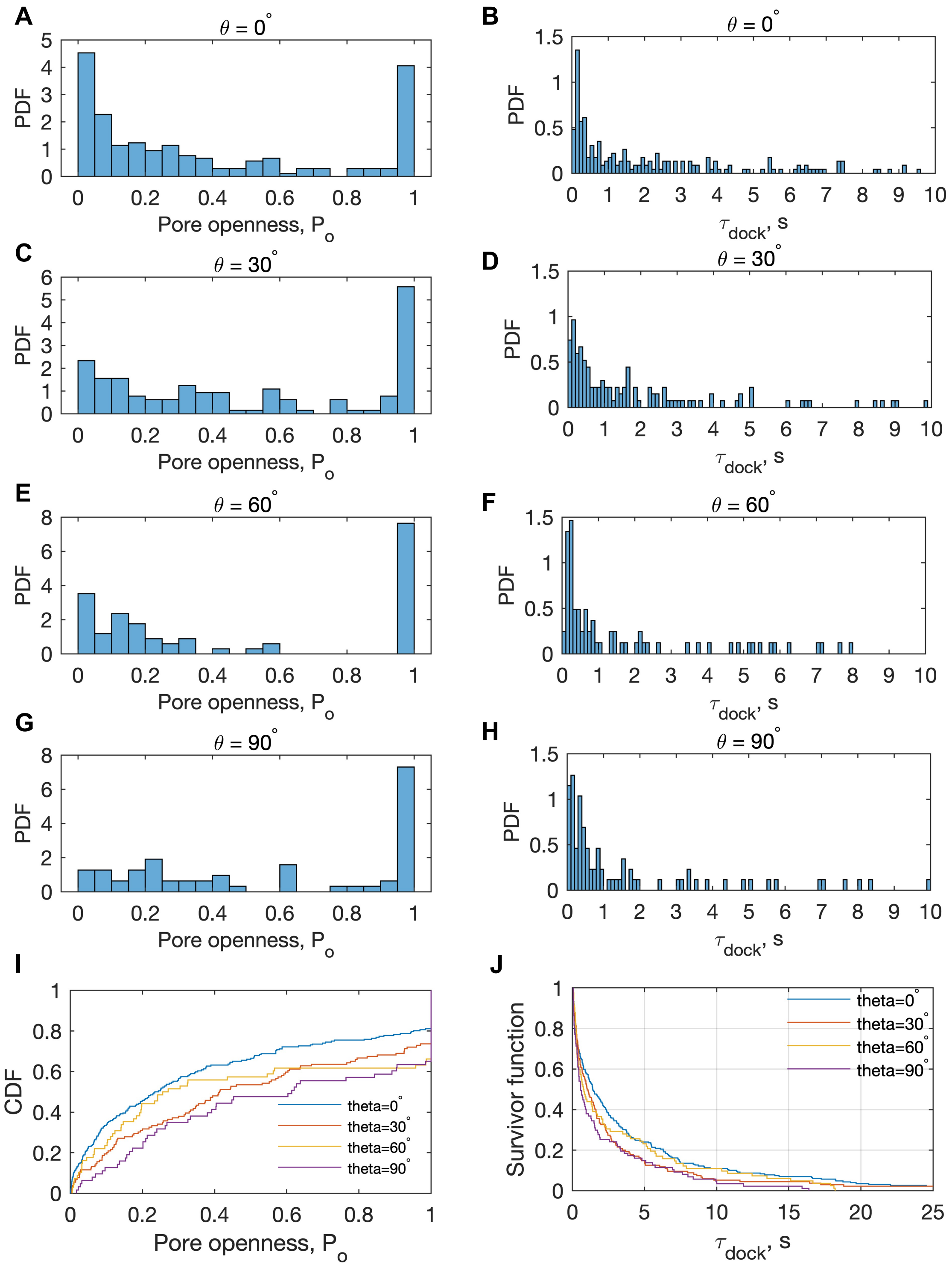

A
θ = 0°
PDF
Pore openness, P_o
B
θ = 0°
PDF
τ_{dock}, s
C
θ = 30°
PDF
Pore openness, P_o
D
θ = 30°
PDF
τ_{dock}, s
E
θ = 60°
PDF
Pore openness, P_o
F
θ = 60°
PDF
τ_{dock}, s
G
θ = 90°
PDF
Pore openness, P_o
H
θ = 90°
PDF
τ_{dock}, s
I
CDF
Pore openness, P_o
theta=0°
theta=30°
theta=60°
theta=90°
J
Survivor function
τ_{dock}, s
theta=0°
theta=30°
theta=60°
theta=90°

Figure S6
